# CRISPR/Cas9-based generation of knockdown mice by intronic insertion of artificial microRNA using longer single-stranded DNA

**DOI:** 10.1038/srep12799

**Published:** 2015-08-05

**Authors:** Hiromi Miura, Channabasavaiah B Gurumurthy, Takehito Sato, Masahiro Sato, Masato Ohtsuka

**Affiliations:** 1 Department of Molecular Life Science, Division of Basic Medical Science and Molecular Medicine, Tokai University School of Medicine, 143 Shimokasuya, Isehara, Kanagawa 259-1193, Japan; 2 Mouse Genome Engineering Core Facility, Department of Genetics, Cell Biology and Anatomy, University of Nebraska Medical Center, Omaha, NE, 68198, USA; 3 Department of Immunology, Division of Basic Medical Science and Molecular Medicine, Tokai University School of Medicine, 143 Shimokasuya, Isehara, Kanagawa 259-1193, Japan; 4 Section of Gene Expression Regulation, Frontier Science Research Center, Kagoshima University, 8-35-1 Sakuragaoka, Kagoshima, Kagoshima 890-8544, Japan; 5 The Institute of Medical Sciences, Tokai University, 143 Shimokasuya, Isehara, Kanagawa 259-1193, Japan

## Abstract

Knockdown mouse models, where gene dosages can be modulated, provide valuable insights into gene function. Typically, such models are generated by embryonic stem (ES) cell-based targeted insertion, or pronuclear injection, of the knockdown expression cassette. However, these methods are associated with laborious and time-consuming steps, such as the generation of large constructs with elements needed for expression of a functional RNAi-cassette, ES-cell handling, or screening for mice with the desired knockdown effect. Here, we demonstrate that reliable knockdown models can be generated by targeted insertion of artificial microRNA (amiRNA) sequences into a specific locus in the genome [such as intronic regions of endogenous *eukaryotic translation elongation factor 2* (*eEF-2*) gene] using the Clustered Regularly Interspaced Short Palindromic Repeats/Crispr associated 9 (CRISPR/Cas9) system. We used *in vitro* synthesized single-stranded DNAs (about 0.5-kb long) that code for amiRNA sequences as repair templates in CRISPR/Cas9 mutagenesis. Using this approach we demonstrate that amiRNA cassettes against exogenous (eGFP) or endogenous [*orthodenticle homeobox 2* (*Otx2*)] genes can be efficiently targeted to a predetermined locus in the genome and result in knockdown of gene expression. We also provide a strategy to establish conditional knockdown models with this method.

The study of gene functions in mice is generally achieved by disrupting the gene to generate a knockout model, and the animal is subjected to phenotypic analysis to understand the effects of *complete* loss of a given protein. Studying the effects of *intermediate* levels of protein loss can also provide valuable insights into gene function during development and disease, especially for those genes where complete protein loss results in embryonic lethality. Over the past decade, several transgenic knockdown models have been generated by expressing short hairpin RNA (shRNA), or artificial microRNA (amiRNA) to undertake gene dose effect studies^1^,^2^,^3^. In such knockdown models, the target sequences against RNA interference (RNAi) are designed as complementary strands so that they bind to mRNA for its degradation or inhibit translation.

Knockdown mice expressing shRNA or amiRNA are generated by injection of a transgenic cassette into the pronucleus of zygotes^4^,^5^,^6^, which then gets inserted into the genome randomly, or by embryonic stem (ES) cell-based methods that target the transgene of a specific locus in the genome^7^,^8^. The former method often causes variegated transgene expression due to reasons such as local effects of the integration site, transgene silencing, adverse effects due to multiple integration sites, and/or multiple tandem transgene insertions. Although the ES cell-targeting approach can overcome such pitfalls, this method is laborious, time-consuming, and cost-prohibitive. Additionally, in both the strategies, a typical transgenic construct constitutes several kilobase pair (kbp) sequence of various elements such as, a suitable promoter, amiRNA sequence often coupled with a reporter gene and a polyA signal.

Endogenous miRNAs are often contained within the introns of protein-coding genes. The intronic miRNAs are co-expressed along with the host genes. The presence of miRNA in an intron does not seem to affect the expression of the host gene, unlike those situated in the 3′ untranslated regions (3′ UTRs) of genes^9^. Thus, we anticipate that knockdown mice can be obtained by insertion of amiRNA sequence into the intronic regions of an endogenous gene, without affecting expression of the host gene. If such a strategy (of inserting amiRNA into intronic regions) indeed results in reliable knockdown, it could provide numerous intronic sites in the genome to serve as choices for targeted insertion of amiRNA sequences.

The CRISPR/Cas9 system has emerged as a method of choice to rapidly edit the genomes of cells and organisms using the Non-Homologous End-Joining (NHEJ) pathway or the Homology-Directed Repair (HDR) pathway^10^,^11^. The HDR pathway is used for inserting DNA of interest (DOI) at the cut site by co-introducing repair-template DNA either as single-stranded DNA (ssDNA) or double-stranded DNA (dsDNA). While the ssDNA repair template requires shorter homology arms and is inserted at a very high efficiency, the dsDNA repair template requires long homology arms and is inserted with much lower efficiency^12^,^13^,^14^. Due to limitations in the overall length of ssDNA that can be synthesized, the ssDNA repair approach cannot be used to insert longer sequences. The current length limit of commercially synthesizable ssDNA oligonucleotides is up to 200-bases. Further, based on the literature, there are no reports that have used ssDNA longer than 200-bases for targeted modification of the genome.

Here, we utilized a two-step method to synthesize longer ssDNA molecules and demonstrate that they efficiently serve as repair templates for CRISPR/Cas9-mediated knocking-in of sequences. In the first step, RNA was synthesized from a DNA template. In the second step, reverse transcription of the RNA was performed to generate ssDNA. We synthesized ssDNA coding for amiRNA sequences against exogenous (eGFP) and endogenous [*orthodenticle homeobox 2* (*Otx2*)] genes and used them as repair templates in CRISPR/Cas9-mediated knocking-in experiments. We demonstrate that ssDNA templates encoding amiRNA sequences were inserted at high efficiency into intron 6 of *eukaryotic translation elongation factor 2* (*eEF-2*), which resulted in successful knockdown of the genes. By combining the Cre-*lox*P system with this approach, we also demonstrate that the inserted amiRNA sequences can be conditionally expressed. Furthermore, unlike the complex designs essential in random- or the ES cell-based targeted transgenesis approaches, this system enables the use of shorter, less complex amiRNA knockdown transgenic cassettes. The lengths of ssDNA sequences ranged from 296 to 514-bases. Thus, sequences up to 514-bases can be readily synthesized by the method we described here and they can serve as HDR templates to create knock-in mutations using the CRISPR/Cas9 system.

## Results

### Preparation of ssDNA

**Figure 1:**
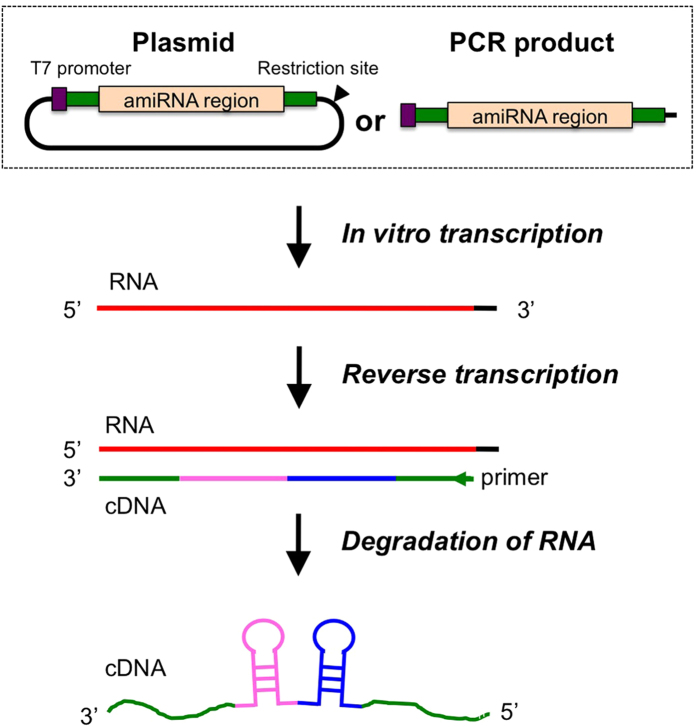
ssDNA synthesis by *iv*TRT strategy. Schematic of plasmid or PCR product templates showing elements such as T7 promoter (purple) and amiRNA fragment flanked by 3′ and 5′ homologous sequences to the target (55-bp each, green), used for *in vitro* transcription of RNA. Plasmid templates are linearized by digesting with a suitable restriction enzyme. *In vitro* transcription is performed using T7 promoter and T7 RNA polymerase. cDNA (ssDNA) is synthesized by reverse transcription and the RNA template is degraded by RNaseH.

We hypothesized that similar to single-stranded oligo-based short sequence insertions, longer ssDNA can insert at a higher efficiency if such longer ssDNA can be synthesized and supplied as repair templates in CRISPR/Cas9 experiments. Because commercially available ssDNA synthesis methods can generate oligonucleotides of only up to 200-bases, we employed an alternative strategy to synthesize longer ssDNA molecules to use them as repair templates. For this purpose, DNA templates transcribed into RNA were first created; then, the RNA was reverse transcribed back to generate ssDNA molecules. We termed this method “*in vitro* Transcription and Reverse Transcription (*iv*TRT)”. The DNA templates were either PCR products or plasmids that contained a T7 promoter, an amiRNA region, and short homology arms for targeted insertion (Fig. 1). Approximately 100 to 500 ng (for PCR product) or 1,250 ng (for plasmid) of gel-purified linear DNA templates were used for RNA synthesis in an *in vitro* transcription reaction. Typical yields ranged from 8 to 18 μg of RNA. Then, 5 μg of RNAs were subjected to cDNA synthesis by reverse transcription reaction. After complete degradation of RNA by RNaseH treatment, resultant cDNA was gel-purified and eluted in microinjection buffer. The yield of cDNAs ranged from 310 to 807 ng that were sufficient for CRISPR/Cas9 injections as the final concentration used for microinjection was 14–20 ng/μl.

### Targeted insertion of amiRNA sequence against eGFP into intronic regions of *eEF-2* gene

**Figure 2:**
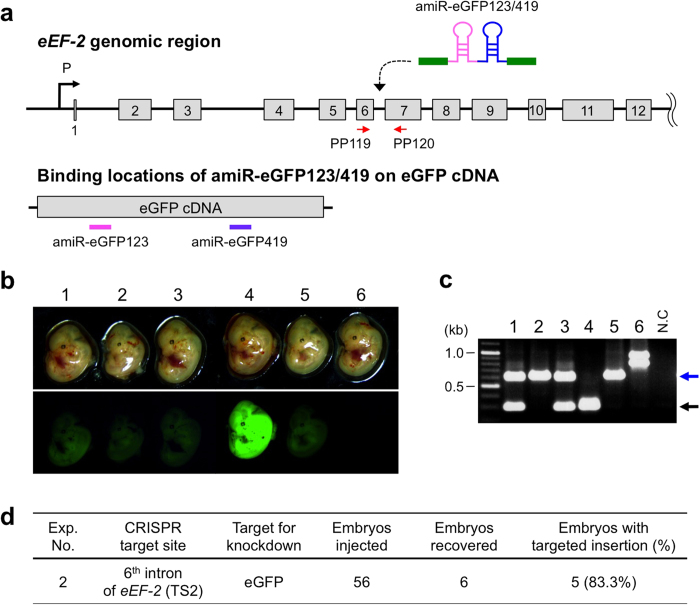
Targeted insertion of ssDNA encoding anti-eGFP amiRNA by CRISPR/Cas9 system (Exp. 2). (**a**) Schematics of targeted integration of amiR-eGFP123/419 sequences into the intron 6 of *eEF-2* gene (upper panel) and location of amiR-eGFP123 and -eGFP419 target sites on eGFP cDNA (lower panel). From the correctly targeted *eEF-2* locus, amiRNAs get transcribed and subsequently bind to the target sites (amiR-eGFP123 = pink line and amiR-eGFP419 = purple line) on eGFP mRNA, leading to eGFP knockdown. Red arrows indicate the location of primer set (PP119/PP120) used for detection of fetuses with targeted insertion. (**b**) eGFP fluorescence among E13.5 day fetuses observed under a fluorescent stereomicroscope showing successful knockdown in some fetuses (see text for details). (**c**) Genotyping of fetuses by PCR using primer set shown in (**a**). The embryo numbers in (**b**) and (**c**) correspond with each other. Expected fragment sizes: wild-type = 301-bp (black arrow), targeted insertion = 625-bp (blue arrow). (**d**) Targeted insertion efficiency. Fetuses containing functional amiRNA sequence were considered as ‘embryos with targeted insertions’ even though insertions were not fully accurate.

We aimed to insert the amiRNA sequences at a genomic site that would readily allow transgene expression without inhibitory positional effects that occur in certain chromosomal locations. Introns of *eukaryotic translation elongation factor 2* (*eEF-2*) gene were selected as candidates because *eEF-2* is expressed at high levels ubiquitously and consistently both during developmental and adult stages^15^. The corresponding intronic regions of mouse and human *eEF-2* genes were aligned and compared, which revealed that parts of mouse introns 1 and 6 were among the evolutionally non-conserved regions. We reasoned that such non-conserved regions are less likely to contain regulatory sequences and therefore not have biological functions. The two less conserved intronic regions in *eEF-2* gene were named Target Site 1 and 2 (TS1 and TS2) (Fig. 2a, Supplementary Fig. S1a). The sgRNAs were designed against TS1 and TS2, as described in Methods section, to insert amiRNA cassettes into these sites. As a first model to test this, we inserted amiRNA to knockdown eGFP gene, using our previously generated eGFP Tg mouse model that shows highly stable eGFP expression^16^,^17^. The 434-base ssDNA copies for TS1 and TS2, containing amiR-eGFP123/419, were prepared and used as repair DNAs (20 ng/μl) in microinjections that contained Cas9 mRNA (10 ng/μl) and respective CRISPR sgRNAs (10 ng/μl). These injection experiments for TS1 and TS2 were designated as Exp. 1 and Exp. 2, respectively. The zygotes used for injection were obtained by *in vitro* fertilization of eggs collected from wild-type C57BL/6 mice using sperm collected from homozygous eGFP Tg mice. Injected zygotes were transferred to oviducts of pseudo-pregnant mice and allowed to develop until embryonic day 13.5 (E13.5), when they were collected and examined for eGFP fluorescence.

Embryos exhibiting low eGFP fluorescence were obtained from both Exp. 1 and Exp. 2, suggesting that the amiRNA sequences effectively knocked down eGFP expression (Fig. 2b, Supplementary Fig. S1b). Genotyping analyses by PCR to detect insertion of amiRNA sequences at the target sites correlated well with the diminished eGFP expression (Fig. 2c, Supplementary Fig. S1c). The insertion efficiency was 50.0% (3/6) and 83.3% (5/6) for Exp. 1 (into TS1) and Exp. 2 (into TS2), respectively (Fig. 2d, Supplementary Fig. S1d). Surprisingly, amiR-eGFP were inserted at both the alleles in Exp. 2 (samples #2, #5 and #6), although the sizes of PCR fragments in Exp. 2 sample #6 were larger than expected (Fig. 2c). Sequencing revealed that Exp. 2 samples #1, #2, #3, and #5 showed correct insertions (Supplementary Fig. S2), whereas the Exp. 2 sample #6 showed anomalous insertions^18^. This sample had two different alleles; one allele (lower band in Fig. 2c) contained the amiRNA cassette (one copy each of amiR-eGFP123 and amiR-eGFP419) together with a part of the vector sequence. The other allele (upper band in Fig. 2c) contained one copy of the amiR-eGFP123, *two* copies of amiR-eGFP419, and a part of the vector sequence (Supplementary Fig. S3). Notably, this sample showed near complete loss of eGFP fluorescence, which suggests homozygosity of insertion of amiR-eGFP123-eGFP419 cassette and an extra copy of amiR-eGFP419 (in one of the alleles) may have contributed to more efficient knockdown. However, it is difficult to rule out effect of vector sequences on the expression of the amiRNA cassette. Of note, the PCR-amplified fragments of all the insertion alleles at TS1 region (Exp. 1) showed unexpected sizes (Supplementary Fig. S1c), and sequencing revealed that all the alleles contained partial insertion of the ssDNA cassettes and/or deletions near the TS1 genomic region [e.g., 154-base pair (bp) deletion in the amiR-eGFP123 region was detected in Exp. 1 sample #3; Supplementary Fig. S4]. This suggests that either the TS1 site is unstable during ssDNA-mediated repair or that possible secondary structures in the homology arm regions of ssDNA repair template may have interfered with the correct insertion of the template.

### Targeted insertion of amiRNA sequence against an endogenous gene

We next tested the above strategy to knockdown an endogenous gene, *Otx2,* which encodes for a homeobox-containing transcription factor involved in craniofacial development (i.e., parts of head, brain, and eye)^19^,^20^. Because decreased *Otx2* levels are closely associated with malformation of the head or eye^21^,^22^,^23^, identification of knockdown effects can be readily detected by the morphological phenotype.

**Figure 3:**
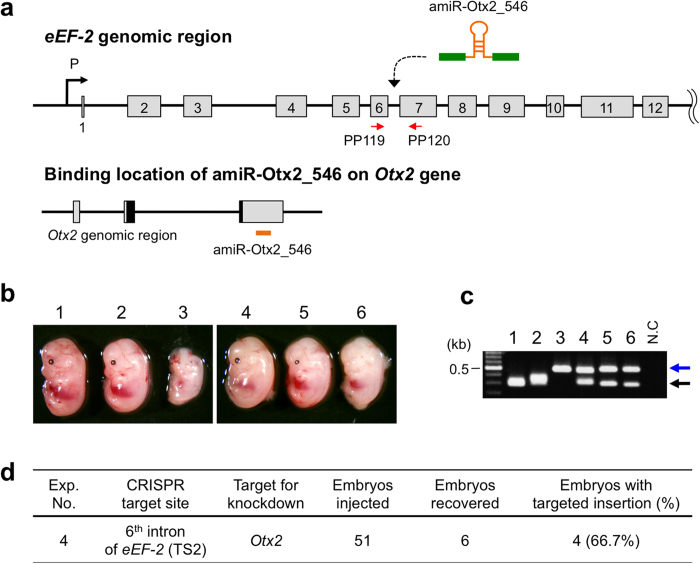
Targeted insertion of ssDNA encoding anti-*Otx2* amiRNA by CRISPR/Cas9 system (Exp. 4). (**a**) Schematics of targeted integration of amiR-Otx2_546 into intron 6 of *eEF-2* gene (upper panel) and *Otx2* genomic region showing amiR-Otx2_546 binding site (lower panel). The exons of *Otx2* are shown as boxes and the boxes with homeodomain region are shaded black. Red arrows indicate the location of primer set (PP119/PP120) used for detection of fetuses with targeted insertion. (**b**) The resultant fetuses photographed at E14.5. (**c**) Genotyping of fetuses by PCR using primer set shown in (**a**). The embryo numbers in (**b**) and (**c**) correspond with each other. Expected fragment sizes: wild-type = 301-bp (black arrow), targeted insertion = 487-bp (blue arrow). (**d**) Targeted insertion efficiency. Fetuses containing functional amiRNA sequence were considered as ‘embryos with targeted insertions’ even though insertions were not fully accurate.

Based on the results of eGFP knockdown, the TS2 site that showed a better rate of insertion of intact sequences was used as the target site for insertion of amiRNA sequences against *Otx2* (Fig. 3a and Supplementary Fig. S5a). Two different amiRNA target sequences against *Otx2* were tested: amiR-Otx2_518 (Exp. 3) and amiR-Otx2_546 (Exp. 4). The ssDNAs for these amiRNA sequences were 296-bases long that were injected at 14 to 20 ng/μl concentration into C57BL/6 zygotes, together with 10 ng/μl of Cas9 mRNA and 10 ng/μl of sgRNA. Injected zygotes were transferred to oviducts of pseudo-pregnant mice. The fetuses were recovered at E14.5 and examined for knockdown phenotypes. We observed putative *Otx2* knockdown phenotypes in two of the embryos derived from Exp. 4 that included amiR-Otx2_546 in the injection mix (Fig. 3b). One embryo (Exp. 4 sample #3) exhibited clear reduction in head, eye, and body size. This phenotype partially resembled *Otx2* conditional knockout mouse reported by Fossat *et al.* (2006)^23^. The anophthalmia (lack of both eyes) phenotype was observed in the embryo #6 in Exp. 4. This is similar to the *Otx2* hypomorphic (*Otx2*^*AA/AA*^) phenotype reported by Bernard *et al.* (2014)^22^. However, no embryos showing putative *Otx2* knockdown phenotypes were obtained from amiR-Otx2_518 injected fetuses (Supplementary Fig. S5b).

The genotyping revealed that the insertion efficiency was 10.0% (1/10) and 66.7% (4/6) for Exp. 3 and Exp. 4, respectively (Fig. 3c and Supplementary Fig. S5c). Consistent with the observed phenotypes, amiRNA sequence was inserted into both alleles of *eEF-2* intron 6 in the Exp. 4 sample #3 fetus (Fig. 3b,c and Supplementary Fig. S6). In addition, the Exp. 4 sample #6 embryo that exhibited anophthalmia also contained the expected amiR-Otx2_546 insertion in the genome (Fig. 3b,c). However, Exp. 4 samples #4 and #5 and Exp. 3 sample #10 fetuses did not exhibit obvious knockdown phenotypes. Sequence analysis revealed that Exp. 4 sample #4 fetus lacked 23-bp nucleotides from the cassette, which included part of the 3′ end of the amiRNA sequence (Supplementary Fig. S7) that was not thought to be critical for amiRNA processing. On the other hand, Exp. 4 sample #5 and Exp. 3 sample #10 had intact amiR-Otx2. The observation that knockdown did not occur in these fetuses suggests that the amiRNA sequences are inefficient for knockdown and/or low proportion of inserted cells (mosaicism) in these embryos. Sequence analyses of multiple bands appeared in Exp. 4 samples #1 and #2 showed that these bands contained *indel* mutations and did not contain ssDNA-derived sequences. Taken together, these results indicate that insertion of amiRNA sequence into the *eEF-2* intron 6 can cause knockdown of an endogenous gene and result in observable phenotypes.

### Targeted insertion of conditional knockdown amiRNA sequences

**Figure 4:**
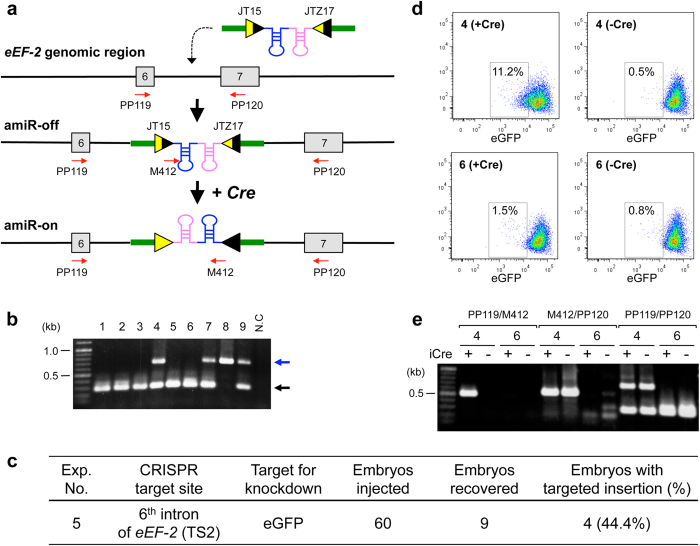
Targeted insertion of ssDNA encoding Cre-activatable anti-eGFP amiRNA by CRISPR/Cas9 system (Exp. 5). (**a**) Schematics of targeted integration of reverse orientated ‘amiR-eGFP123/419’ and mutant *lox*P sites (*JT15* and *JTZ17*) into the intron 6 of *eEF-2* gene and subsequent Cre-*lox*P recombination to switch the amiRNA cassette to the right orientation. Functional amiRNAs do not get produced from the targeted ‘*amiRNA-off*’ allele because of the opposite orientation of amiRNA with respect to the *eEF-2* gene. After Cre recombination, the allele gets converted to ‘*amiRNA-on*’ that produces functional amiRNA. Red arrows indicate the primers (PP119, PP120 and M412) used for genotyping. (**b**) Genotyping of fetuses by PCR using primer set (PP119/PP120). Expected fragment sizes: wild-type = 301-bp (black arrow), targeted insertion = 705-bp (blue arrow). (**c**) Targeted insertion efficiency. (**d**) Dotplot of embryonic feeder cells, derived from Exp. 5 samples #4 (upper) and #6 (lower), nine days after transfection with (left) or without (right) the iCre plasmid. The cells showing weak eGFP fluorescence are partitioned within the box in each plot. (**e**) Genotyping of embryonic feeder cells used in the experiment (**d**) by PCR with primer sets shown in (**a**).

The constitutive expression of certain amiRNA sequences that leads to embryonic lethality can eventually result in unavailability of a model for further studies^24^,^25^. A conditional expression strategy offers the best solution in such cases to generate a viable model. Hence, we next tested applicability of Cre-*lox*P system, a widely used conditional activation system, in our knockdown strategy. For this purpose, mutant *lox*P sequences *JT15* and *JTZ17* were included to flank the amiRNA sequence, as shown in Fig. 4a, and the cassette was placed in the opposite direction to *eEF-2* gene orientation. Because of the opposite orientation, the functional amiRNA will not be produced, and the allele will be in the ‘off’ state (i.e., ‘*amiRNA-off*’ allele). After Cre administration, Cre-*lox*P-mediated recombination occurred between inversely-oriented *JT15* and *JTZ17*^26^ that resulted in inversion of amiRNA region to convert the allele to ‘on’ state (‘*amiRNA-on*’ allele), which will allow for expression of functional amiRNA driven by the *eEF-2* promoter. Because *JT15* and *JTZ17* contain mutations within their inverted-repeat regions, the inversion step will be unidirectional and the ‘*amiRNA-on*’ state will get locked after Cre recombination^27^.

To test this concept, a 514-base long ssDNA was synthesized containing amiRNA against eGFP, along with flanking mutant *lox*Ps and homology regions for targeted integration. This ssDNA (20 ng/µl) was injected into zygotes along with Cas9 mRNA (10 ng/μl) and sgRNA (10 ng/μl) (Exp. 5). The zygotes were obtained as described in Exp. 1 and Exp. 2. E14.5 fetuses derived from injected zygotes were recovered and analyzed by PCR to detect insertion of amiRNA sequences at the target sites. Four out of nine embryos that recovered (44.4%) had expected insertion, of which one showed insertion into both alleles (Fig. 4b,c). Sequence analyses revealed that all four fetuses contained the correct insertion allele (e.g., Exp. 5 sample #8; Supplementary Fig. S8).

To test if the engineered knockdown cassette can undergo conversion from *‘amiRNA-off’* to *‘amiRNA-on’* state upon Cre recombination, the iCre expression vector (pAYC) was transfected into fibroblasts isolated from the embryos (Exp. 5 sample #4 as a test sample and sample #6 as negative control) and assessed eGFP expression. The eGFP fluorescence intensities were examined by fluorescence-activated cell sorting (FACS) after culturing transfected cells for nine days, which showed that a moderate reduction of eGFP florescence occurred only in the iCre-transfected cells derived from Exp. 5 sample #4 embryo. In contrast, no florescence reduction was noted in un-transfected control and amiRNA-negative embryo-derived cells (Exp. 5 sample #6; Fig. 4d). Consistent with this result, PCR genotyping using primer set PP119/M412 detected the presence of *‘amiRNA-on’* allele generated by Cre-*lox*P-mediated inversion only in the iCre-transfected sample (Exp. 5 sample #4; Fig. 4e).

## Discussion

The CRISPR/Cas9 system has emerged as a popular genome editing method because of its technical simplicity. It is used not only for gene disruption, but also for targeted modification using HDR. The ssDNA donors, up to 200-bases long, are used as donors for insertion of short stretches^10^. Because longer ssDNAs (>200-bases long) cannot be commercially synthesized, longer insertions require the use of dsDNAs (plasmid-based constructs). In this study, we synthesized ssDNA donors of about 0.5-kb long, using a technique called *iv*TRT. The ssDNA donors were then used in CRISPR/Cas9-mediated targeted insertion experiments. We observed up to 83.3% overall insertion efficiency, and up to 50% insertion efficiency in both alleles. Notably, the homology arm lengths in these samples were only 55-bases on each side. These results suggest that ssDNA-based insertion is efficient even though the length of donors was about 0.5-kb. It would be interesting to evaluate this strategy for longer ssDNA molecules of kilobases longer. Considering that *in vitro* transcription reactions can typically generate over 4- to 5-kb long RNAs, and up to 10-kb long cDNA can be synthesized using certain reverse transcriptase, *iv*TRT could be used for synthesizing ssDNA of kilobases long.

If indeed our method can be applied for synthesis and insertion of longer ssDNA templates, the method will be very useful for inserting larger expression cassettes such as promoter:cDNAs:termination signals or fusing protein tags (e.g., GFP or Cre preceded by self-cleaving peptidases such as T2A to the 3′ end of last codons of genes). It will be of interest to systematically assess the efficiencies of ssDNA donors of varying lengths. Longer ssDNAs may require longer homology arms for efficient targeting. However, this is not expected to be the case considering that successful HDR can occur with as few as 50 to 60-bases for ssDNA termini. We are currently testing insertion efficiencies of longer ssDNAs generated with *iv*TRT and also some of the applications described above. It is also likely that secondary structures in certain longer sequences result in lower insertion efficiency and/or inaccurate insertion. Future studies will be able to systematically evaluate such parameters.

Among the total of 14 samples that contained insertion at TS2 site (that includes both eGFP and *Otx2* amiRNA cassette insertions), 12 samples (86%) had correctly inserted cassettes and only 2 samples (Exp. 2 sample #6 and Exp. 4 sample #4) contained inaccurate insertions. Exp. 2 sample #6 contained vector-derived sequences in addition to the intact or extra amiRNA sequences (Supplementary Fig. S3). The presence of vector-derived sequences is probably due to incomplete removal of template DNA by DNase treatment after the *in vitro* transcription reaction during the ssDNA preparation method and the residual dsDNA (that contained vector sequences) may have got inserted at the cut site. We also observed, a 23-bp deletion near the 3′ end of amiRNA region in one sample (Exp. 4 sample #4; Supplementary Fig. S7).. This could be due to insertion of partially degraded cDNA (lacking 3′ downstream of amiRNA region) or loss of the terminal 23 nucleotides of the ssDNA template during insertion. It is likely that this deletion would not have occurred due to incomplete cDNA synthesis because this terminus originated from the primer end. The fact that about 86% (12 out of 14) of the offspring had correctly inserted donor sequences suggest that the ssDNA synthesized through *iv*TRT method can generate fairly high accuracy insertion of knock-in cassettes using CRISPR/Cas9.

The presence of vector-derived sequences in Exp. 2 sample #6 fetus indicated a possibility that the residual dsDNA (incompletely removed during ssDNA preparation step) could have served as a donor template for double-strand break (DSB) repair. In order to independently test this possibility, dsDNA (supplied as PCR product) was injected and the samples were analyzed for insertion efficiency of dsDNA template. A total of 20% (2/10) zygotes obtained from dsDNA injection showed targeted insertion, while 50% (12/24) of zygotes from ssDNA injection contained insertion allele (Supplementary Figure S9). It is noteworthy that dsDNA injection (at 20 ng/μl concentration) caused excessive damage to zygotes compared to ssDNA injection at least in these experimental conditions. We thus conclude that dsDNA-based targeted insertion with short homology arms is also possible, although ssDNA knock-in strategy seems to be more efficient than the dsDNA knock-in strategy. It should be noted that linear dsDNA can readily get inserted at random sites (as a transgene). Therefore, dsDNA will not be an ideal repair template compared to using it as a circular dsDNA or as a linear ssDNA template.

We were skeptical about the suitability of the novel genetic locus (*eEF-2*), and the intronic targeting approach, for effectiveness of amiRNA-mediated knockdown because the successful expression of inserted amiRNA cassettes would depend on the normal expression of the host gene, and that the insertion event should also not affect the host gene expression. The efficiencies of knockdown of both endogenous (*Otx2*) and exogenous (eGFP) genes suggest that the *eEF-2* locus that we selected is a good choice, and the inronic site insertion offers as a novel strategy. The knockdown effects in fetuses, however, seemed variegated in both the eGFP and *Otx2* knockdown, which could be attributed to the insertions being mono-allelic or bi-allelic, and/or mosaic. Indeed, higher knockdown was observed in samples showing bi-allelic insertions of amiRNAs against eGFP as well as *Otx2*. Although variegations of knockdown effects can get stabilized once the mutations are established by germline transmission, phenotypic variations observed in F0 animals could often provide valuable information considering that there are human diseases that occur due to mosaic somatic mutations^28^.

The insertion of cassettes into the intronic sites may affect correct transcription and splicing of the host gene. In order to rule out this possibility, we examined if TS2 site insertions affected splicing of *eEF-2* gene in cultured cells. As shown in Supplementary Figure S10, the splicing of the *eEF-2* gene was intact. Further, we recently generated viable pups that contained conditional amiRNA against *Otx2* gene, and these animals did not show any abnormalities (data not shown). Analysis using the GENSCAN Web Server at MIT (http://genes.mit.edu/GENSCAN.html)^29^ was performed to rule out whether intronic insertion of the cassette could affect prediction of splicing events. The results suggested that the modified *eEF-2* genomes (obtained in Exp. No. 2, 4 and 5) predicted normal splicing patterns (Supplementary data). These results indicate that insertion of amiRNA sequences into the *eEF-2* introns did not have any effects on its transcription and splicing. It is likely that other introns (or introns of other genes) may not be similar to the one tested here in terms of their suitability to insert foreign sequences, particularly if they contain any regulatory sequences.

To overcome the limitations associated with constitutive knockdown, such as lack of tissue-specificity or lethality due to RNAi, conditional approaches using Cre-*lox*P or inducible RNAi using tetracycline have been utilized^24^,^25^. In a Cre-*lox*P-based conditional RNAi approach, to prevent generation of constitutively active shRNA, *lox*P-flanked stuffer sequences were inserted into the shRNA loop or between the promoter and the shRNA^24^,^25^. In this case, RNAi is induced after Cre-mediated excision of stuffer sequence from the cassette. As an alternative method, Stern *et al.* (2008) used irreversible inversion strategy with two pairs of mutant *lox*P sites (*lox2272* and *lox5171*), by which RNAi is induced after inversion of amiRNA from antisense to sense orientation^30^. We were able to readily adopt the inversion strategy using mutant *lox*Ps (*JT15* and *JTZ17*) in our conditional knockdown approach unlike the floxed stuffer sequence strategy, which cannot be applied here, because the presence of stuffer sequence in the transcription unit may disrupt proper transcription of the host gene. AmiRNA-based knockdown was observed when the iCre-expression vector was introduced into embryonic fibroblast cells prepared from the knock-in fetuses, indicating that conditional activation of the knockdown cassettes inserted at the *eEF-2* locus operate as expected. It should be noted that the inversion strategy may not be used in intronic regions that code for overlapping transcripts from the antisense strand.

In conclusion, this study successfully establishes a simple and efficient method of generation of knockdown mice using CRISPR/Cas9 system. To generate donor repair DNA encoding amiRNA sequences, we used ssDNAs synthesized by a simple strategy called *iv*TRT and achieved a high integration efficiency. We also demonstrate that amiRNA targeted to an intronic site (e.g., intron 6 of *eEF-2* gene) confer knockdown effects in fetuses, indicating that this strategy is powerful for rapid and feasible analyses of developmentally important genes. We are currently investigating the heritability of the knockdown phenotype among the animal models generated by this approach. To our knowledge, this is the first report which demonstrates that successful knockdown models can be generated by targeted insertion of amiRNA sequences to intronic sequences. It will be interesting to examine introns of other genes as potential candidates for achieving desired levels of higher/lower expression or even tissue-specific expression of the inserted amiRNA cassette. The method described here does not require construction of complex vectors and can be accomplished through a direct microinjection method, without using the laborious ES cell-based steps. Our method offers a simple, fast, and efficient means to generate knockdown models that are useful for hypomorphic analysis of gene function. Further, this method can be used for insertion of longer stretches of knock-in sequences that cannot be accomplished using commercially synthesized single-stranded oligonucleotides.

## Methods

### Designing of amiRNA

The amiRNA sequences for targeting eGFP (eGFP123 and eGFP419) or the *Otx2* (Otx2_518 and Otx2_546) gene were designed using BLOCK-iT RNAi designer (Invitrogen, Carlsbad, CA). For amiRNA sequences against eGFP, two pre-miRNA sequences (eGFP123 and eGFP419) were assembled in tandem, whereas the amiRNAs against *Otx2* were individually tested (either Otx2_518 or Otx2_546).

### Synthesis of ssDNA by *iv*TRT

The synthetic DNA oligonucleotides (top and bottom; Supplementary Table S1) were heat denatured and annealed prior to cloning into a vector supplied in the “BLOCK-iT Pol II miR RNAi Expression Vector Kit with EmGFP” (Invitrogen, Carlsbad, CA). The regions spanning pre-miRNA(s) were amplified by PCR with primer sets containing 55-bases of homology arms (PP109/PP110 for Exp. 1, PP111/PP112 for Exp. 2, Exp. 3 and Exp. 4) using KOD-Plus-Neo DNA polymerase (TOYOBO, Osaka, Japan), and were cloned into *Sma*I site of pUC119 vector. The regions containing the amiRNA and homology arm sequences were re-amplified from sequence-confirmed clones by PCR (with primer sets: PP123/M272 for Exp. 1, PP125/M322 for Exp. 2 and Exp. 4, PP125/M272 for Exp. 3; Supplementary Table S1) using KOD-Plus-Neo DNA polymerase and the DNA fragments were gel-purified and used as templates for *iv*TRT. The template for *in vitro* transcription in Exp. 5 (pP170 containing “T7 promoter – 5′ homology arm – JT15 – amiR-eGFP419 – amiR-eGFP123 – JTZ17 - 3′ homology arm – *Nco*I site”) was generated by gene synthesis (GENEWIZ, Inc., South Plainfield, NJ) ligation-based cloning, and used in an *in vitro* transcription after digestion with *Nco*I. The RNAs complementary to the DNA fragments were *in vitro* synthesized with mMESSAGE mMACHINE T7 Ultra transcription kit (Ambion, Austin, TX) and purified using MEGAclear kit (Ambion) after DNase treatment as described previously^10^. The cDNAs were reverse transcribed from synthesized RNAs using SuperScriptIII Reverse Transcriptase (Invitrogen) using primer (PP124 for TS1 and PP126 for TS2). The cDNAs were gel-purified and the concentrations were measured using a NanoDrop^TM^ 2000. To avoid clogging during microinjection, ssDNAs were filtered by passing through an Ultrafree-MC filter (HV; 0.45 μm pore size; #UFC30HV00; Millipore, Billerica, MA).

### Preparation of sgRNA and Cas9 mRNA

sgRNAs against TS1 and TS2 in *eEF-2* gene were designed using CRISPR design^5^,^14^ and CHOPCHOP^31^. The sgRNAs listed as highly potential targets in both the programs but not located in the putative branching site in the intron were picked. The templates for sgRNA synthesis were PCR amplified with primer sets (PP105/M939 for TS1, and PP106/M939 for TS2) using pUC57-sgRNA vector (addgene number: #51132)^32^ as template. Four hundred ng of gel-purified PCR products were subjected to RNA synthesis with MEGAshortscript™ T7 Kit (Ambion) and DNase treatment followed by purification of mRNA using MEGAclear Kit. *Xba*I-digested pBGK^10^ was used as a template for synthesizing Cas9 mRNA. The synthesis and purification of Cas9 mRNA was performed as described for RNA synthesis steps of ssDNA synthesis.

### Mice

The C57BL/6 mice, used as embryo donors for both eGFP and *Otx2* knockdown experiments, were obtained from CLEA Japan, Inc. (Tokyo, Japan). eGFP Tg mouse line (B6.Cg-Gt(ROSA)26Sor<tm2.1(CAG-EGFP)Maoh>), used as embryo donor for eGFP knockdown experiments that has a single copy eGFP transgene integrated at the *Rosa26* locus, was previously generated in our facility^17^. All mice were maintained in the Center of Genetic Engineering for Human Diseases (CGEHD) animal facility at Tokai University School of Medicine in Japan. All the animal experiments were performed in accordance with institutional guidelines and were approved by The Institutional Animal Care and Use Committee at Tokai University (Permit Number: #143037).

### Microinjection into one-cell mouse embryos

ssDNA, sgRNA and Cas9 mRNA were mixed (at concentrations of 14–20 ng/μl for ssDNA, 10 ng/μl for sgRNA and 10 ng/μl for Cas9 mRNA) and co-injected into both the pronuclei and cytoplasm of fertilized eggs obtained using *in vitro* fertilization. Fertilized eggs derived from eGFP Tg male and C57BL/6 female for eGFP knockdown and from C57BL/6 male and female for *Otx2* knockdown were used. Injected eggs were cultured overnight in KSOM medium at 37 °C with 5% CO_2_, and resulting two-cell embryos were transferred into the oviducts of pseudo-pregnant ICR females.

### Detection of targeted insertion of amiRNA sequences

The fetuses were isolated from pseudo-pregnant mice at E13.5 or E14.5 that were implanted with injected zygotes. Targeted ssDNA insertion was assessed by observing under a fluorescence microscope (only for eGFP knockdown), PCR-based genotyping, and sequencing. Expression of eGFP fluorescence in fetuses was checked using the Leica M165 FC (Leica, Wetzlar, Germany) with filter sets for GFP. The primer sets used for PCR-based genotyping were as follows: PP113/M412 in Exp. 1; PP119/PP120 in Exp. 2, Exp. 3, Exp. 4 and Exp. 5.

Direct sequencing for some samples was performed using the gel-purified or Exo-SAP-treated PCR products as templates. For some PCR-amplified bands, fragments were cloned into a pUC119 or a TA Cloning vector (TOPO® TA Cloning Kit, Life Technologies), and the insert sequences were determined. See Supplementary Table S2 for details.

### Preparation of primary embryonic fibroblast cells

E14.5 fetuses were isolated in sterile conditions and rinsed with phosphate buffered saline (PBS) containing antibiotics. The livers and heads were dissected out and the remaining parts were chopped into small pieces and were digested with 0.1% Trypsin/EDTA for 20 min at 37 °C. Culture medium was then added to the samples and were filtered with nylon mesh to obtain well dispersed population of cells. The cells were centrifuged, re-suspended in culture medium, counted and plated on 6 cm culture plate, cultured until they became confluent, trypsinized, centrifuged, re-suspended in cell culture freezing medium with DMSO and frozen at −80 °C for future use.

### Cre-*lox*P recombination in primary fibroblast cells and FACS analysis

The embryonic primary fibroblasts were plated onto 6 cm plates. The cells were electroporated, two days after plating, with a Neon^TM^ transfection system (Invitrogen) according to the manufacturer’s recommendation. In each electroporation, 5 × 10^4^ cells were electroporated with 500 ng iCre-expression plasmid (pAYC) using the following electroporation conditions; 1350 V pulse voltage, 30 ms pulse width and one pulse. After the electroporation, each sample was plated onto a 24-well plate containing culture medium and cultured for 9 days and was subjected to FACS analysis to assess eGFP fluorescence.

Cells were washed twice with PBS and incubated with 0.05% trypsin-EDTA at 37 °C in 5% CO_2_ incubator for 5 min, culture medium was added to the plates to stop cell dissociation and then passed through a 35-μm mesh filter (BD Biosciences, Franklin Lakes, NJ, USA) for FACS analysis. The cells were subjected to flow cytometry using an LSRFortessa (BD Biosciences) to assess eGFP fluorescence. Data were analyzed using FlowJo software (Tree Star, Inc., Ashland, OR, USA) and % cells showing eGFP loss indicative of Cre-induced activation of knockdown was quantified.

The Cre-*lox*P recombination in the cells was further confirmed by PCR with primer sets: PP119/M412, M412/PP120 and PP119/PP120 using the genomic DNA isolated from them.

## Additional Information

**How to cite this article**: Miura, H. *et al.* CRISPR/Cas9-based generation of knockdown mice by intronic insertion of artificial microRNA using longer single-stranded DNA. *Sci. Rep.* **5**, 12799; doi: 10.1038/srep12799 (2015).

## Supplementary Material

Supplementary Information
