# Supplementary material for: CRISPR/Cas9-based generation of knockdown mice by intronic insertion of artificial microRNA using longer single-stranded DNA
Source: Sci Rep. 2015 Aug 5;5:12799. doi: 10.1038/srep12799 (PMC4525291; doi:10.1038/srep12799)
Supplement: Supplementary Information [file srep12799-s1.pdf]

Supplementary Information

CRISPR/Cas9-based generation of knockdown mice by intronic insertion of artificial microRNA using longer single-stranded DNA

Hiromi Miura, Channabasavaiah B Gurumurthy, Takehito Sato, Masahiro Sato, Masato Ohtsuka

Supplementary Tables

Supplementary Table S1

Supplementary Table S1 l Oligos used in the present study.

Name	Nucleotide sequence 5'-3'
M272	CAGGAAACAGCTATGACC
M322	TGCGCAACTGTTGGGAAG
M381 (eGFP123_top)	TGCTGATGAACTTCAGGGTCAGCTTGGTTTTGGCCACTGACTGACCAAGCTGACTGAAGTTCAT
M382 (eGFP123_bottom)	CCTGATGAACTTCAGTCAGCTTGGTCAGTCAGTGGCCAAAACCAAGCTGACCCTGAAGTTCATC
M383 (eGFP419_top)	TGCTGTGTAGTTGTACTCCAGCTTGTGTTTTGGCCACTGACTGACACAAGCTGGTACAACTACA
M384 (eGFP419_bottom)	CCTGTGTAGTTGTACCAGCTTGTGTCAGTCAGTGGCCAAAACACAAGCTGGAGTACAACTACAC
M411	GGAATTCTAGTGAGTCGACCAGTGGATC
M412	GGAATTCTGGGTCTAGATATCTCGAGTGC
M939	AAAAAAAGCACCGACTCGG
PP101	CAGCAAAACTACAGGTTATTATTGCTTGTGATCCGCCTCGGAGTATTTTCCATCGTAGTGAGTCGACCAGTGGATC
PP102	AAGGATCTCAAGCAGGAGAGTATAAAACTCGGGTGAGCATGTCTTTAATCTACCTTGGGTCTAGATATCTCGAGTGC
PP103	GTAATACGACTCACTATAGGGCAGCAAAACTACAGGTTATTATTGC
PP104	AAGGATCTCAAGCAGGAGAGTA
PP105	TAATACGACTCACTATAGGGCTACCGGAAAGGGCCCGGAGTTTTAGAGCTAGAAATAGCAAG
PP106	TAATACGACTCACTATAGGGGGAGCGACTTCCCAAGGCTGTTTTAGAGCTAGAAATAGCAAG
PP109	TGACAGCCTGTAGGGCTGGCTTCCTTGGACACCTCCAGTGGGCTGAACGCCTTCCTAGTGAGTCGACCAGTGGATC
PP110	TGCTCACGTGTGAACTCAGATAGAAAACAGAGACGGGGGCTACCGGAAAGGGCCCTGGGTCTAGATATCTCGAGTGC
PP111	TCCTGGACCCCATCTTCAAGGTGGGTAAGCTGTGGTCCTGGCCTGTGTTCCCAGCTAGTGAGTCGACCAGTGGATC
PP112	GTTTGCACAAGGGAAGCAGATGAGCAAGGCCAGGTCATGGGAGCGACTTCCCAAGTGGGTCTAGATATCTCGAGTGC
PP113	ATATCTGCGCGTCCCTGA
PP114	TGAGGCTCAAGTCACATAACC
PP115	TTGTGCTTGGTGATGTGG
PP116	CACACACCTCTTCCCCAAA
PP117	TGGGGAGACCGGTGAGTA
PP118	GAGGCCCCTCGTATAGCA
PP119	GCCAATGGCAAGTTCAGTA
PP120	GCCTTGAGCAATGGCTTG
PP123	GTAATACGACTCACTATAGGGTGACAGCCTGTAGGGCTG
PP124	TGCTCACGTGTGAACTCAGATA
PP125	GTAATACGACTCACTATAGGGTCCTGGACCCCATCTTCA
PP126	GTTTGCACAAGGGAAGCAGA
PP130 (Otx2_518_top)	TGCTGTATAGGTCATGGGATAGGACCGTTTTGGCCACTGACTGACGGTCCTATCATGACCTATA
PP131 (Otx2_518_bottom)	CCTGTATAGGTCATGATAGGACCGTCAGTCAGTGGCCAAAACGGTCCTATCCCATGACCTATAC
PP132 (Otx2_546_top)	TGCTGTAGCCTTGACTATAACCTGAAGTTTTGGCCACTGACTGACTTCAGGTTAGTCAAGGCTA
PP133 (Otx2_546_bottom)	CCTGTAGCCTTGACTAACCTGAAGTCAGTCAGTGGCCAAAACTTCAGGTTATAGTCAAGGCTAC
PP164	CACCGTAGGCTTTGGTTCTG

Supplementary Table S2

Supplementary Table S2 | Sequencing of PCR products amplified from modifid genome

Exp.	Fetus	Band	Strategy for sequencing	Primers used for sequencing	Note
1	#1		sequencing after cloning of PCR product	M13 reverse	
1	#3		direct sequencing of PCR product	PP113	Supplementary Figure. S4
1	#5		sequencing after cloning of PCR product	M13 reverse	
2	#1		sequencing after cloning of PCR product	PP120	
2	#2		direct sequencing of PCR product	PP120	
2	#3		sequencing after cloning of PCR product	PP120	
2	#5		direct sequencing of PCR product	PP120	Supplementary Figure. S2
2	#6	upper	sequencing after cloning of PCR product	PP119, PP120	Supplementary Figure. S3
2	#6	lower	direct sequencing of PCR product	PP119, PP120	Supplementary Figure. S3
3	#10		sequencing after cloning of PCR product	M13 reverse	
4	#3		direct sequencing of PCR product	PP120	Supplementary Figure. S6
4	#4		sequencing after cloning of PCR product	M13 reverse	Supplementary Figure. S7
4	#5		sequencing after cloning of PCR product	M13 reverse	
4	#6		sequencing after cloning of PCR product	M13 reverse	
5	#4		sequencing after cloning of PCR product	PP120	
5	#7		sequencing after cloning of PCR product	PP120	
5	#8		direct sequencing of PCR product	PP119, PP120	Supplementary Figure. S8
5	#9		sequencing after cloning of PCR product	PP120	

Supplementary Figures

Supplementary Figure S1

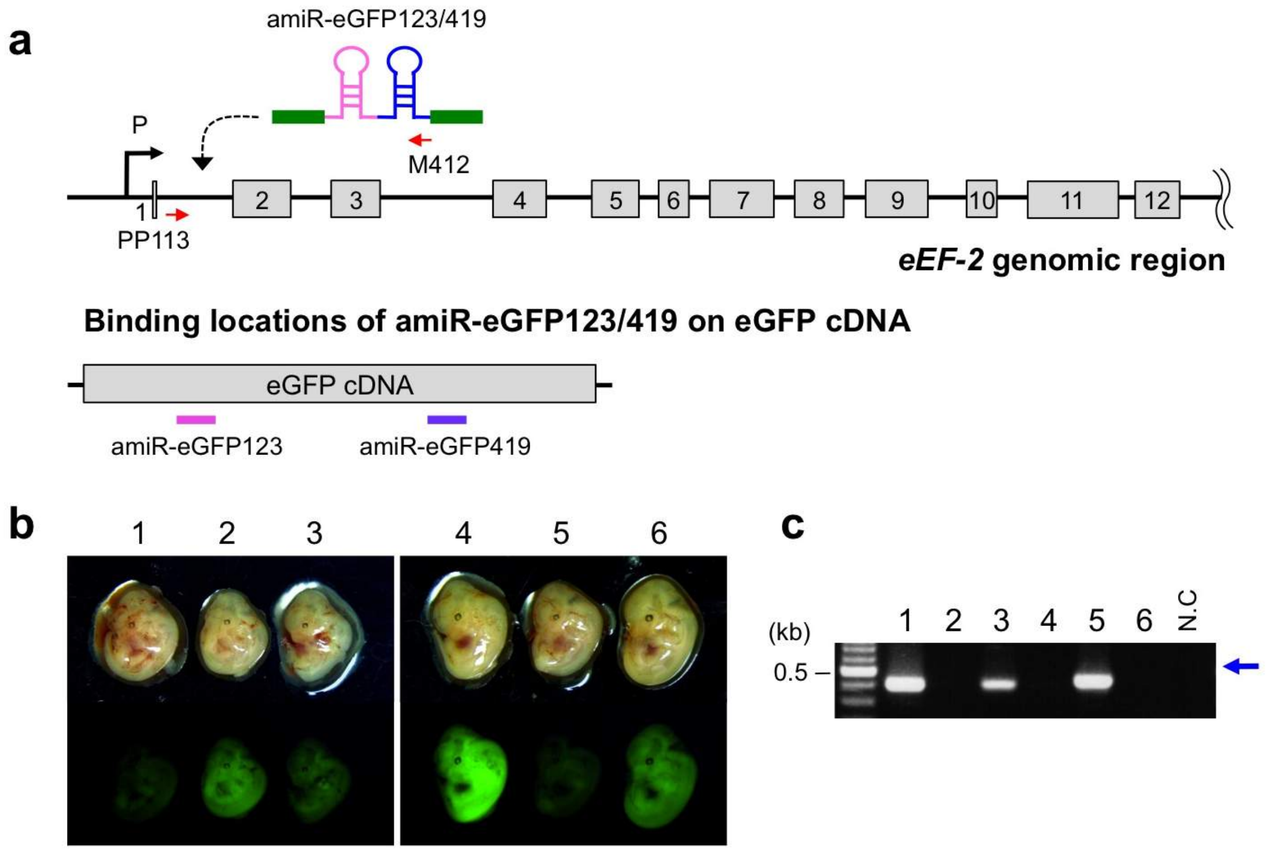

d

Exp. No.	CRISPR target site	Target for knockdown	Embryos injected	Embryos recovered	Embryos with targeted insertion (%)
1	1st intron of *eEF-2* (TS1)	eGFP	50	6	3 (50.0%)

Supplementary Figure S1 | Targeted insertion of ssDNA encoding anti-eGFP amiRNA by CRISPR/Cas9 system (Exp. 1). (a) Schematics of targeted integration of amiRNA-eGFP123/419 into the intron 1 of *eEF-2* gene (upper panel) and location of amiRNA-eGFP123 and -eGFP419 target sites on eGFP cDNA (lower panel). From the correctly targeted *eEF-2* locus, amiRNAs get transcribed and subsequently bind to the target sites (amiR-eGFP123 = pink line and amiR-eGFP419 = purple line) on eGFP mRNA, leading to eGFP knockdown. Red arrows indicate the location of primer set (PP113/M412) used for detection of fetuses with targeted insertion. **(b)** eGFP fluorescence among E13.5 fetuses observed under a fluorescent stereomicroscope showing successful knockdown in some fetuses (see text for details). **(c)** Genotyping of fetuses by PCR using primer set shown in (a). The embryo numbers in (b) and (c) correspond with each other. Expected fragment sizes: targeted insertion = 562-bp (blue arrow). **(d)** Targeted insertion efficiency. Fetuses containing functional amiRNA sequence were considered as ‘embryos with targeted insertions’ even though insertions were not fully accurate.

Supplementary Figure S2

Exp.2-#5

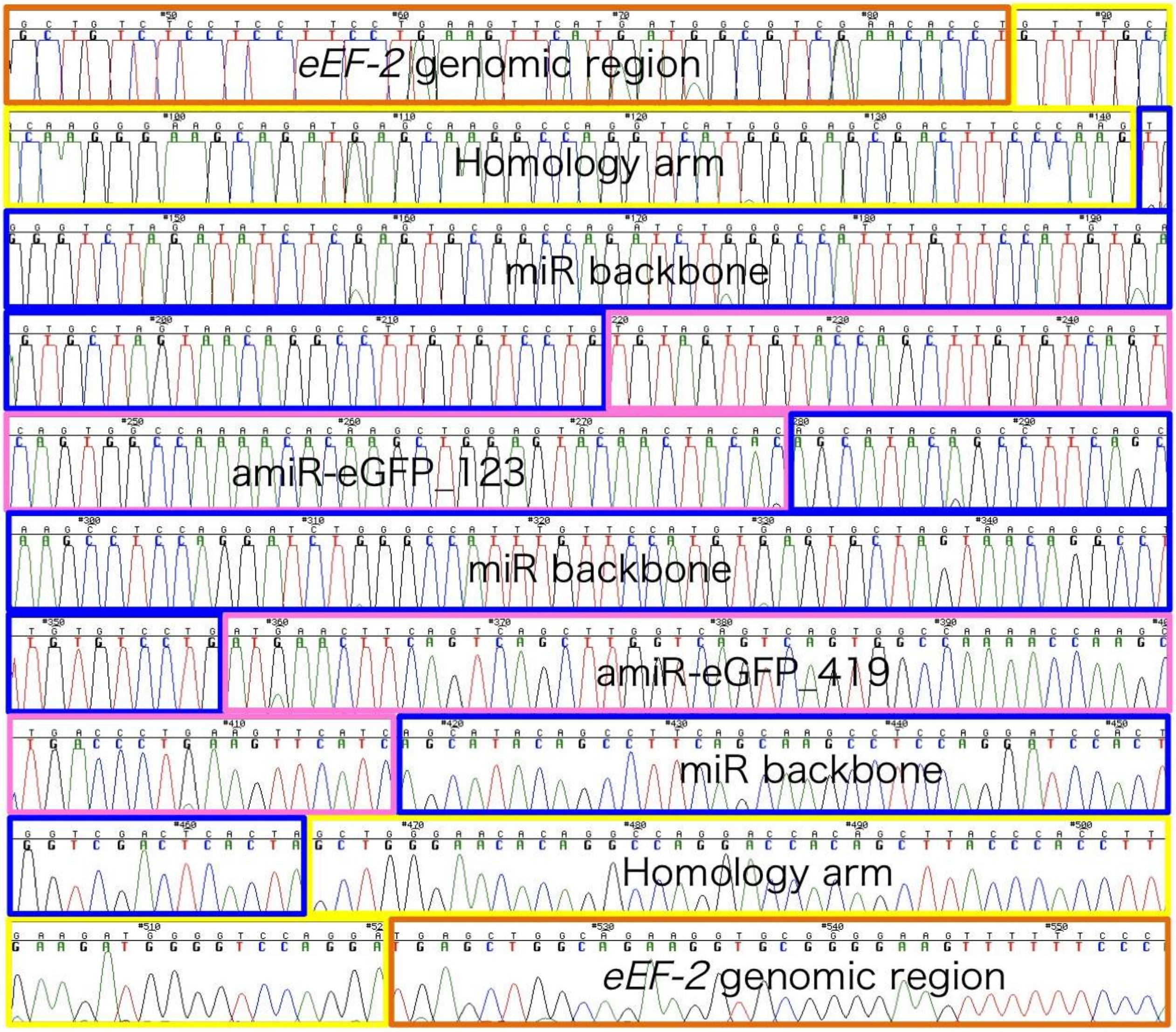

Supplementary Figure S2 | A representative sequencing reaction chromatogram showing correct insertion of ssDNA encoding amiRNAs against eGFP gene at intron 6 of *eEF-2*. The genomic sequence showing insertion derived from Exp. 2 sample #5 is shown.

Supplementary Figure S3

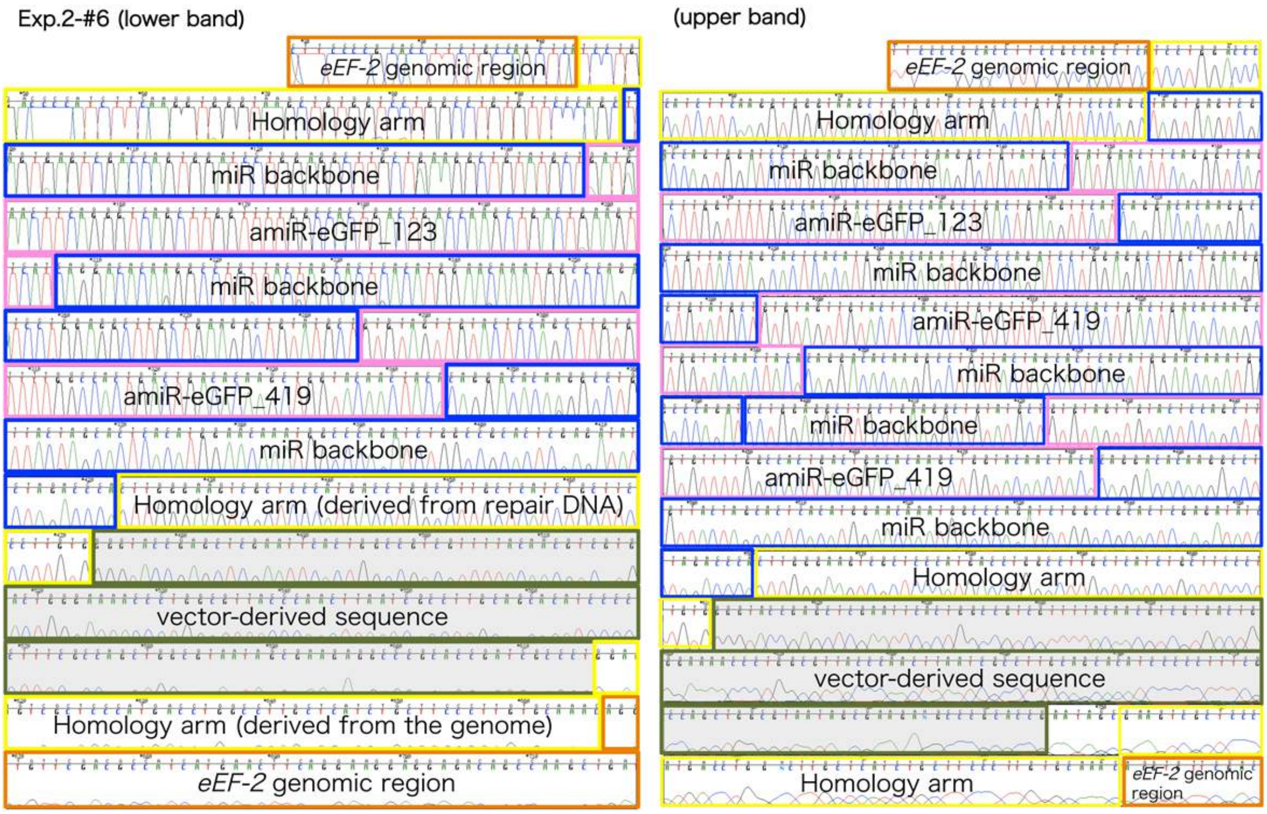

Supplementary Figure S3 | Sequencing reaction chromatograms showing insertion of ssDNA encoding amiRNAs against eGFP gene at intron 6 of *eEF-2*. The inserted genomic sequences (left for lower band and right for upper band) showing insertion derived from Exp. 2 sample 6 is shown. Vector-derived sequence region is shaded in gray.

Supplementary Figure S4

Exp.1-#3

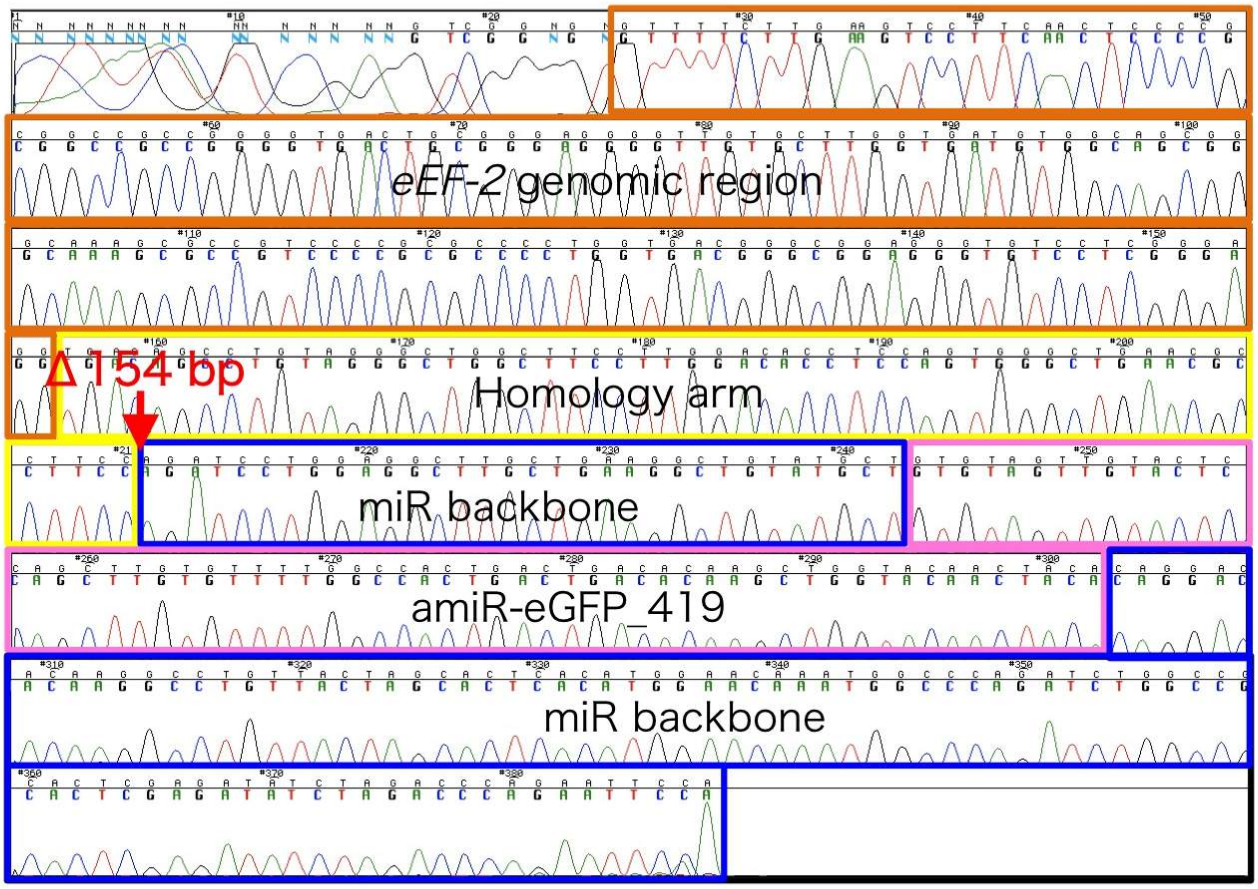

Supplementary Figure S4 | A representative sequencing reaction chromatogram showing insertion of imperfect ssDNA encoding amiRNAs against eGFP gene at intron 1 of *eEF-2*. The inserted genomic sequence showing insertion derived from Exp. 1 sample #3 is shown.

Supplementary Figure S5

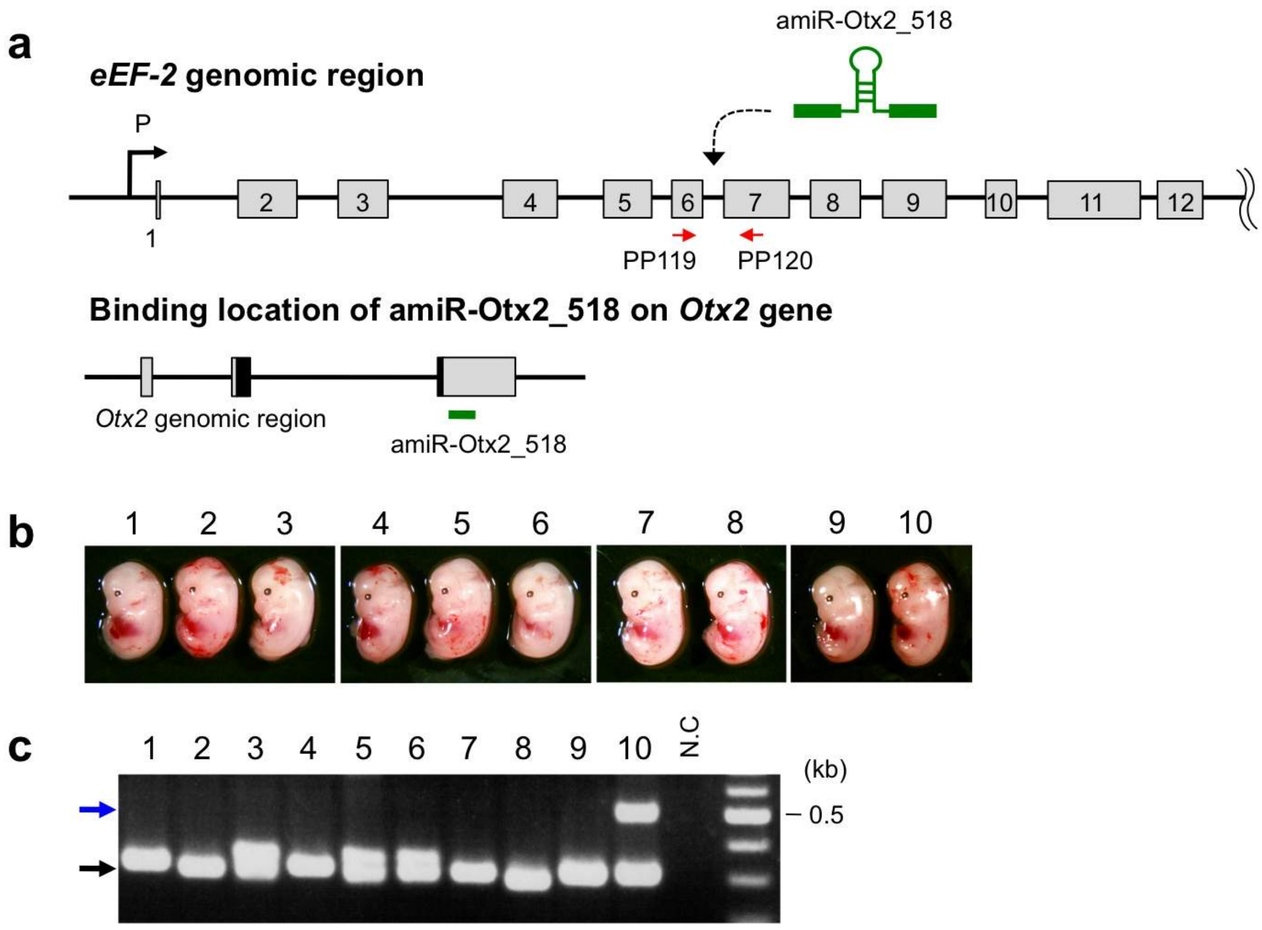

d

Exp. No.	CRISPR target site	Target for knockdown	Embryos injected	Embryos recovered	Embryos with targeted insertion (%)
3	6th intron of *eEF-2* (TS2)	*Otx2*	56	10	1 (10.0%)

Supplementary Figure S5 | Targeted insertion of ssDNA encoding anti-Otx2 amiRNA by CRISPR/Cas9 system (Exp. 3). (a) Schematics of targeted integration of amiR-Otx2_518 into the intron 6 of *eEF-2* gene (upper) and *Otx2* genomic region showing amiRNA-Otx2_518 binding site (lower panel). The exons of *Otx2* are shown as boxes and the boxes with homeodomain region are shaded black. Red arrows indicate the location of primer set (PP119/PP120) used for detection of fetuses with targeted insertion. **(b)** The resultant fetuses photographed at E14.5. **(c)** Genotyping of fetuses by PCR using primer set shown in (a). The embryo numbers in (b) and (c) correspond with each other. Expected fragment sizes: wild-type = 301-bp (black arrow), targeted insertion = 487-bp (blue arrow). **(d)** Targeted insertion efficiency.

Supplementary Figure S6

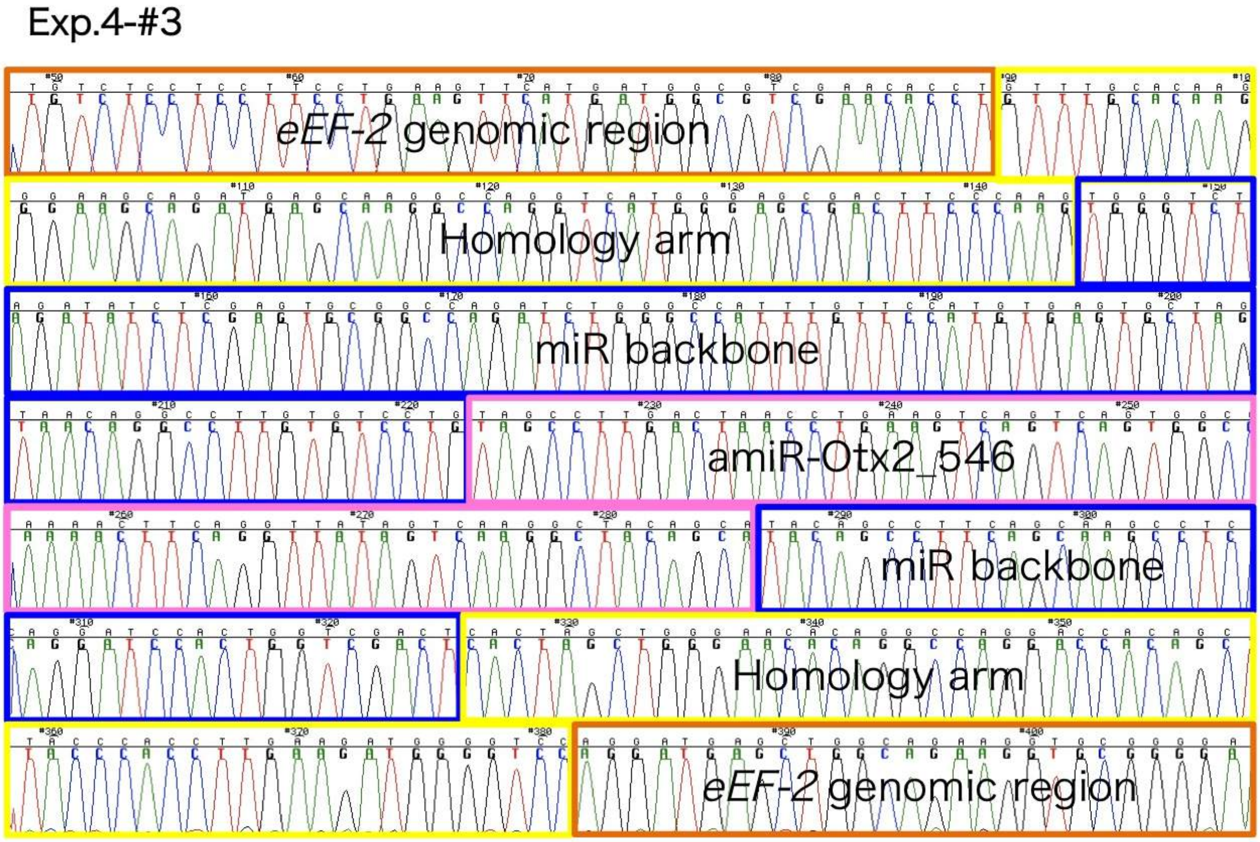

Supplementary Figure S6 | A representative sequencing reaction chromatogram showing correct insertion of ssDNA encoding amiRNAs against *Otx2* gene at intron 6 of *eEF-2*. The genomic sequence showing insertion derived from Exp. 4 sample #3 is shown.

Supplementary Figure S7

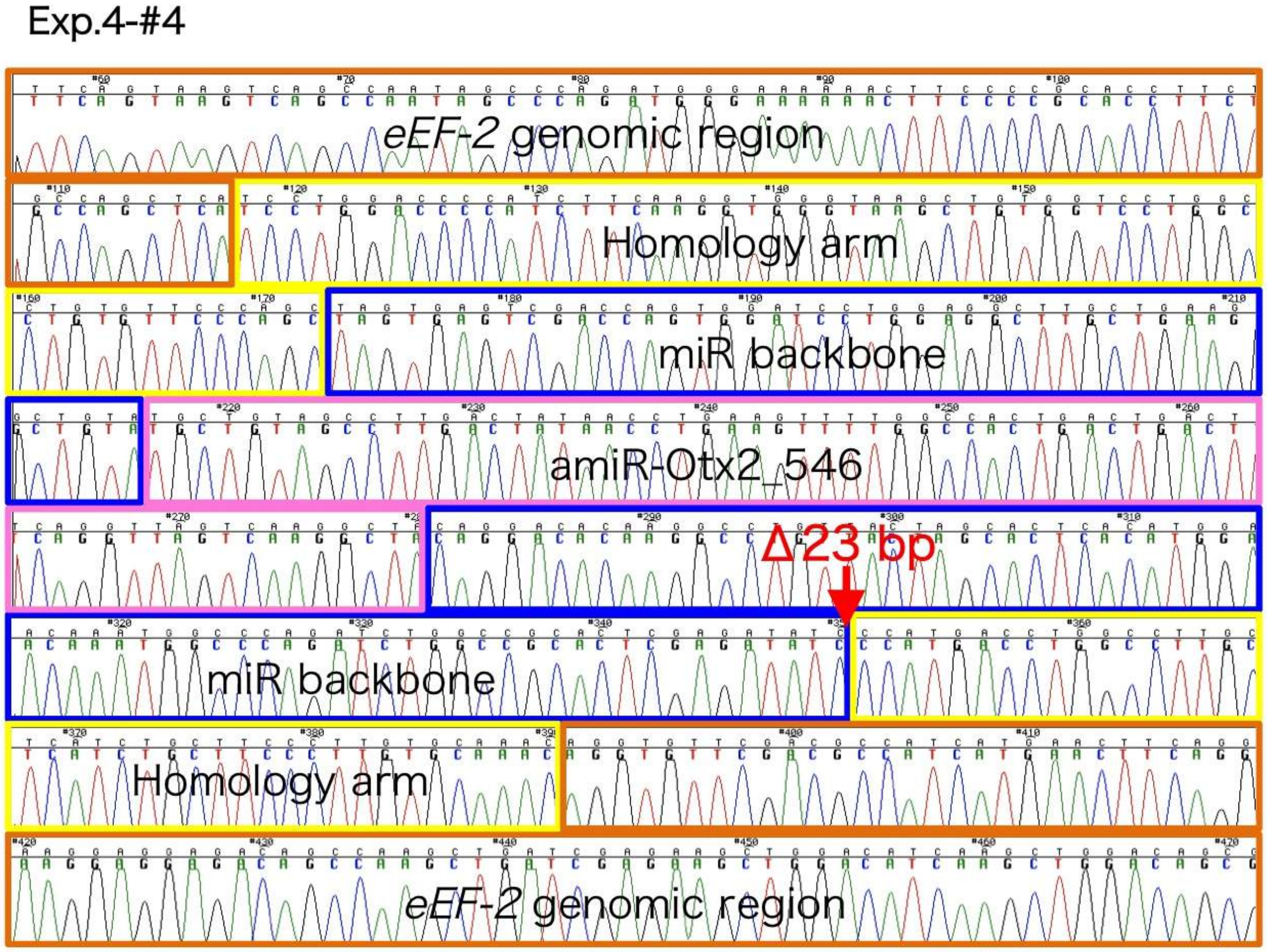

Supplementary Figure S7 | A representative sequencing reaction chromatogram showing insertion of imperfect ssDNA encoding amiRNAs against *Otx2* gene at intron 6 of *eEF-2*. The genomic sequence showing insertion derived from Exp. 4 sample #4 is shown.

Supplementary Figure S8

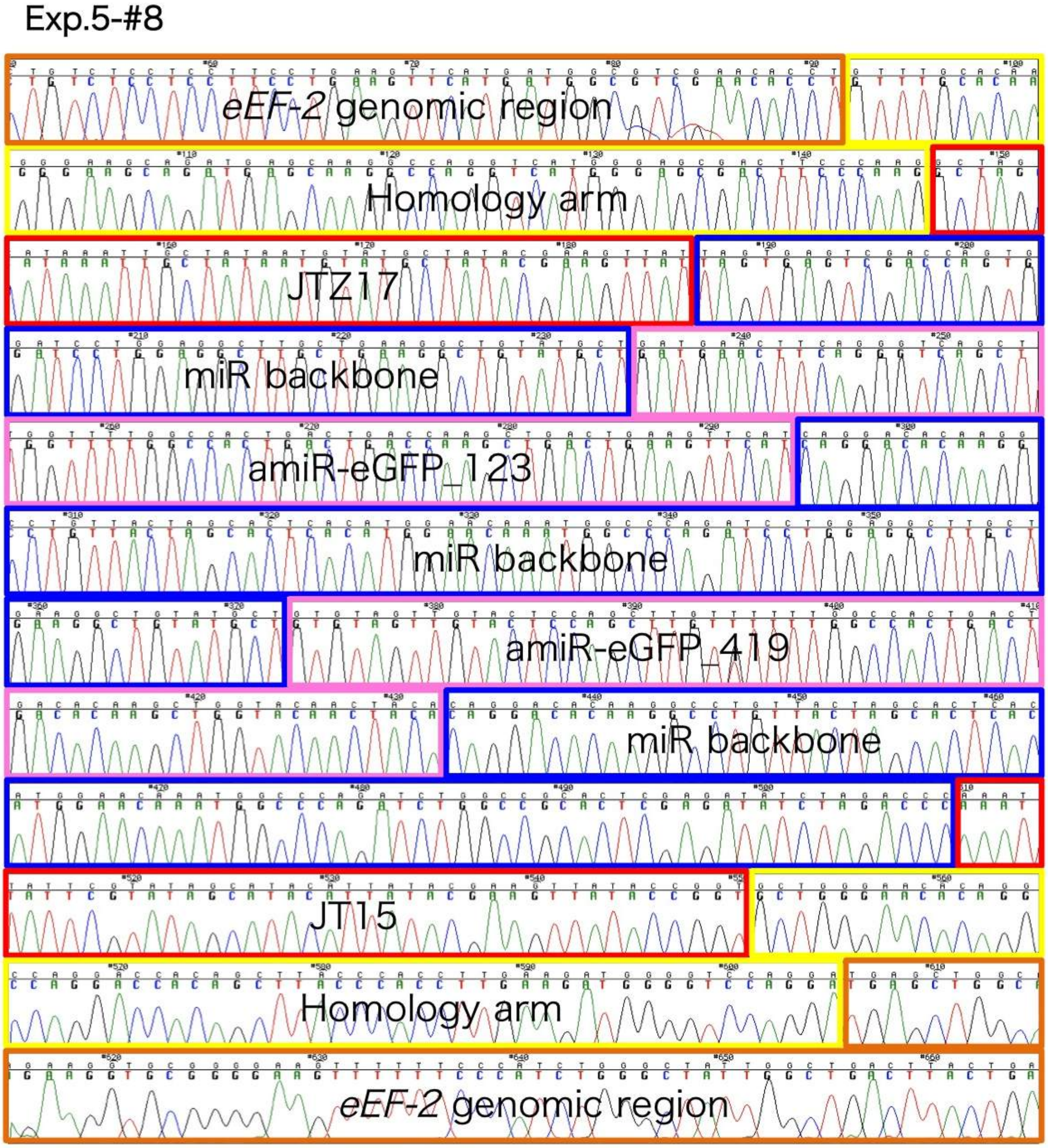

Supplementary Figure S8 | A representative sequencing reaction chromatogram showing correct insertion of ssDNA encoding amiRNAs against eGFP gene at intron 6 of *eEF-2*. The genomic sequence showing insertion derived from Exp. 5 sample #8 is shown.

Supplementary Figure S9

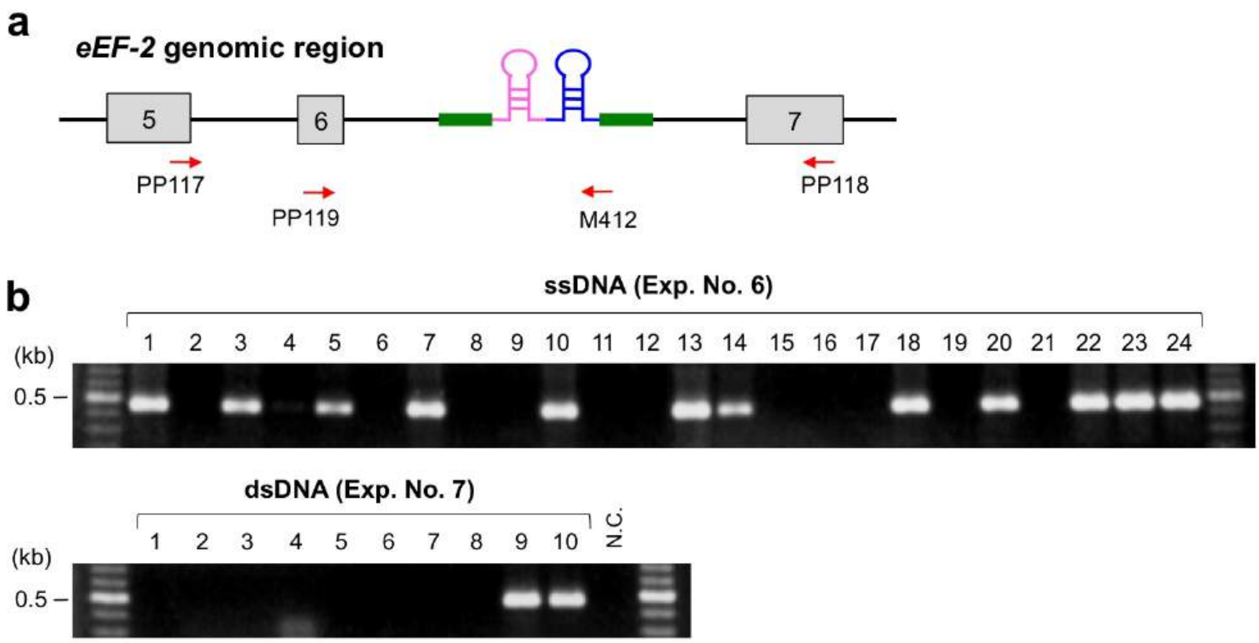

c

Exp. No.	CRISPR target site	Target for knockdown	Donor DNA	Eggs injected	No. of blastocysts developed	Targeted insertion (%)
6	6th intron of *eEF-2* (TS2)	*eGFP*	ssDNA	88	24	12 (50%)
7	6th intron of *eEF-2* (TS2)	*eGFP*	dsDNA	88	10	2 (20%)

Supplementary Figure S9 | Comparison of insertion efficiencies of ssDNA or dsDNA repair templates (encoding anti-eGFP amiRNA) by the CRISPR/Cas9 system (Exp. 6 and 7). (a) Genomic structure of *eEF-2* locus with targeted integration of amiRNA-eGFP123/419. Red arrows indicate the location of primer sets (PP117/PP118 and PP119/M412) used for detection of blastocysts with targeted insertion by nested PCR. ssDNA (in Exp. 6) or dsDNA (in Exp. 7) containing amiR-eGFP123/419 and homology arms with 55-bases long on each side were prepared and used as repair DNAs. Each of the repair DNA (20 ng/µl) was subjected to micro-injections together with Cas9 mRNA (10 ng/µl) and sgRNAs for TS2 (10 ng/µl). **(b)** Genotyping of blastocysts by nested PCR using primer set shown in (a). Expected fragment size: 459-bp. **(c)** Targeted insertion efficiency.

Supplementary Figure S10

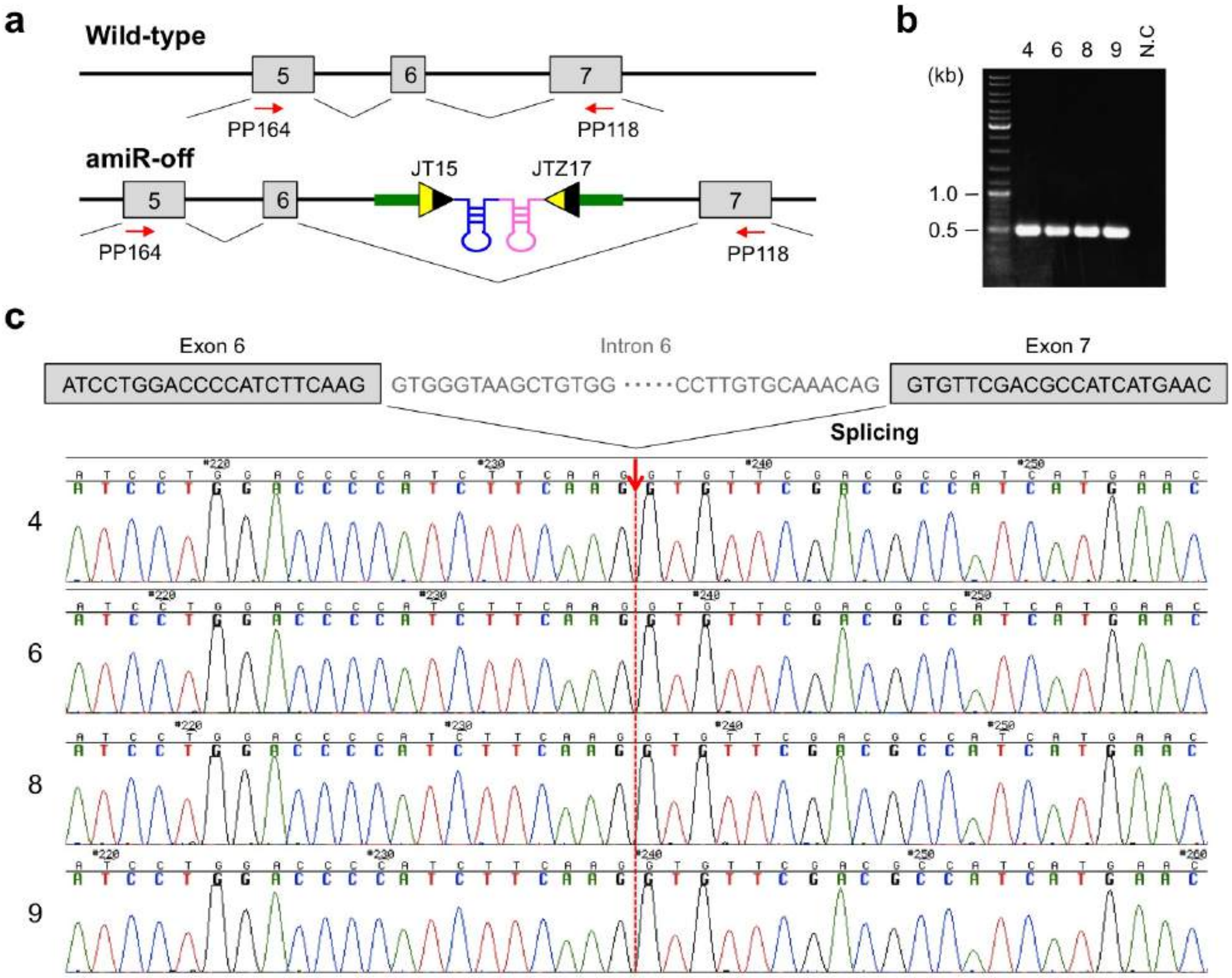

Supplementary Figure S10 | Insertion of sequences in the *eEF-2* intron 6 does not affect its mRNA splicing. (a) Genomic structure and expected splicing pattern of wild-type and '*amiRNA-off*' alleles (containing reverse orientated 'amiRNA-eGFP123/419' and mutant *lox*P sites) of *eEF-2* gene. Red arrows indicate the location of primer set (PP164/PP118) used for reverse transcription PCR (RT-PCR). **(b)** RT-PCR results using cDNAs prepared from embryonic feeder cells derived from the fetuses in Exp. 5 (see Figure 4). The sample numbers in (b) correspond with those in Figure 4b. Sample No.6; wild-type allele, No.4 and 9; heterozygotes for '*amiRNA-off*' allele, No.8; homozygote for '*amiRNA-off*' allele. Expected fragment size: 500-bp (from cDNA). **(c)** Nucleotide sequences of RT-PCR products. Junctional regions between exon 6 and 7 are shown.

Supplementary data

The *eEF-2* genomic sequences (wild-type and modified) and GENSCAN results

The GENSCAN Web Server at MIT (http://genes.mit.edu/GENSCAN.html) was used for analyses.

Coding sequences and inserted sequences (including amiRNA) are shown in red and blue, respectively.

>eEF2_wild-type
GACAAAAAAAGTTTCATGAGAGCGTGGTGTGTTGGCACAGTCTTTTAGTCCGAGCACTGACGCAGTAGATTAAGGCAGATCTTCATG
ATTTGGTGCGGTCCTGGGCTGCATCCAAAGACCTTGTTTCCAAAACAAACAAACGAAAAAGTGTAATAAAAATACAAGCAAGGTTT
TGCAGAAATAAATCTGTGGCCTCAAGGGTCCCTTTCTCCAGCTTTTCCCAGAGGTGTCAAATTACATAACAGTCTCAAACCCAGGAA
ATTGAACTTTGGCACAACCCACTGACCGGTCCGAATTTCAAAGTCTTTTTCCTATTGACCTACAAGGTTTTCAAGAATCATGTTGTAA
GCAACTGTGTTCTGAGGAATCTATGTTTAAAAACCCATCCGTGGATCTTGGCCCAGGGTCCAGAGACTGAGCTAGCCACGCCCCGGC
CGCGCCGCAGCCACTCCCACGGCAGTTCAAGTGTTAAGTCCCAAAGACCGCGCTCTGTGCATGCGCAGACCCGTCCACAGCTGGCTC
CTAGCCAACCCGGCCGGACGAGCACCCGGCGCCGTCACGTGACGCACCCAACCGGCGTCGACCTATAAAAGGCCGGGCGTTGACGT
CAGCGGTCTCTTCCGCCGCAGCCGCCGCCATCGTCGGCGCGCTTCCCTGTTCACCTCTGACTCTGAGAATCCGTCGCCATCCGCCACC
ATGGTGAGTCTCCTCGGCTCCGCTAGACTCGGGGACCGAGACGAATCTCTGGGGCAGCGGGACGTGGCTGTAGCGGGACGCTGAGA
GGGACGGGAGGAAGAGACATGGCTGCCCTGGCCCGGGCGGCAGGACGTGGTCGGGCCGCGGCGCCATATCTGCGCGTCCCTGAGG
GCCTTGGGAGTGTCAACTGCCGAGGTCGGGGTGTTTTCTTGAAGTCCTTCAACTCCCCGCGGCCGCCGGGGTGACTGCGGGAGGGGT
TGTGCTTGGTGATGTGGCAGCGGGCAAAGCGCCGTCCCCGCGCCCCTGGTGACGGGCGGAGGGTGTCCTCGGGAGGTGACAGCCTG
TAGGGCTGGCTTCCTTGGACACCTCCAGTGGGCTGAACGCCTTCCGGGCCCTTTCCGGTAGCCCCCGTCTCTGTTTTCTATCTGAGTT
CACACGTGAGCACCGGTCCCCATAATCTAAGAAAGTGGCTCACTGGGCCTAGTGGCGCATTGTGGCCTTTGATCCGGGCTTTGACCT
TGGCGCACAGCACCCAGTGGTTTGGGGAAGAGGTGTGTGTAGCAGAGGAGGTTTTTTCGTGCTTTGGTCCCAATCAATCCGGCATCT
TTGCAGTGCCGAGGTGGCCGTGCACCTTGGCTTTGAATTCTTGTGCTGAGGTTATGTGACTTGAGCCTCAAGATAGGGTCTTCTAGCA
CAGGCTTGCTCTTAAGTGTCGCAGTTGTCGGTTTCGGCGTTTGTTTAGAGCTGTGGACACATCTGTGAACTTTTGATGCTTATTTCAG
AGGTCCTGGGTTGTACGTTTGAGTCACACTGTGAGCTCAGCTCCAATCTTGGGCCGACATCTGGTTCCTGCCCCTGCTGTGGGGTGCT
ATTGACCCACCGATGCCTGCCAAGTTGGGTTCCCAGAATCAGCCTGGCTGCCCATCCCCCCACCACAGGTGAACTTCACAGTAGATC
AGATCCGTGCCATCATGGACAAGAAAGCCAACATCCGGAACATGTCAGTCATCGCCCATGTGGACCACGGCAAGTCCACGCTGACC
GACTCCCTTGTGTGCAAGGCTGGCATCATTGCCTCTGCCCGAGCTGGGGAGACGCGCTTCACTGACACTCGCAAGGATGAGCAGGAG
CGCTGCATCACAATCAAATCCACGTGAGTGAGGGGACAGCCCCGAGGGGTTGTGCTCTGGGTGTCACTCGGGTGTGTTTGCTGCCAG
TGGACAAATAGTGGCGTGTTTAGGGTCCTCTCTGTGACATGCTTGTGTGTGCTGCCTAGCCTGCCTTGGTCAGATGCTGAATGGACTG
TTGACCTCTTCCTCTCCGTAGCGCCATCTCCCTCTTCTACGAGCTCTCTGAGAACGACCTGAACTTCATTAAGCAGAGCAAGGATGGC
TCGGGCTTCCTCATCAACCTCATCGACTCTCCAGGCCATGTGGACTTCTCTTCAGAGGTGACAGCTGCCTTGCGTGTCACCGATGGAG
CTCTGGTGGTGGTGGACTGTGTGTCTGGTAAGCCTTGGGTGGGAGCTGTAACAGACAGTTGCTGGTGTTTTCATGGCCCAGTACCAC
ATTTTTGGGGGTGTAAATTGAAATCATATTCATTTTAAGGCAAGGATGAATTCTGCTCTAATTTGAAATTGTATGTGCCAGTCCCCAT
GTCAGGGCAGGGAGATTGTGAAGTCCACCTAAGTGTGGGGCCAGTTTCCTATAATTTCAGTGCTCTGAGGCAGAGGTGCATGGGTCT
CTGGAGGTGGGAGGGGCTGAGTGGCATAGGGCCTGGTACACTGGACACACTTGGAGATTTAGCGCCTGGGCATCCATACCGTGTAC
CTTGGAGAGTGCTTGTGTACATTTCACAGACAAAACATACGTGGTCTTCAGGCCTTGAACTGTCAGTGGTTTAGAGTACTTGAGAGT
TAAGGGTAAACACTTAGTCCTTTTCACCCTGTCCTCAGGCGTGTGTGTGCAGACAGAAACTGTGCTGCGCCAGGCCATCGCTGAACG
CATCAAGCCCGTCCTGATGATGAACAAGATGGACCGGGCCCTGCTGGAGCTGCAGCTGGAGCCCGAGGAGCTCTACCAGACCTTCC
AGCGCATCGTGGAGAACGTCAACGTCATCATCTCTACCTACGGCGAGGGCGAGAGTGGGCCCATGGGCAATATCATGGTGCGAAGC
TCTTCTATGGGGGGCGGTCAGGGCCACGCTCCCTGGGAATCTCTGACCCACCATGATGACGGAAATTCTTCACTTTGACCTGATGTCT
GTTGAAGAAACTCAGTGTCTGAGACTGTGGGCTTGTGGCTGGGCTGGCTGCTGACCATCTTGCCGGGCTAACTGCTGCTCTCCTCCCT
CAGATTGACCCCGTCCTGGGCACCGTAGGCTTTGGTTCTGGCCTGCATGGCTGGGCCTTCACCCTGAAGCAGTTTGCGGAGATGTAT
GTGGCCAAGTTTGCAGCCAAGGGTGAGGGCCAGCTGAGCGCAGCCGAGCGTGCCAAGAAAGTAGAGGACATGATGAAGAAGCTGT
GGGGAGACCGGTGAGTACATGGCTGTGTGACTGTCTCTACCCCTGCTACTTGTGGACAATGCCTGCCTGCCGATACTATGTGACTGC
TCTCTACAGGTACTTTGATCCGGCCAATGGCAAGTTCAGTAAGTCAGCCAATAGCCCAGATGGGAAAAAACTTCCCCGCACCTTCTG
CCAGCTCATCCTGGACCCCATCTTCAAGGTGGGTAAGCTGTGGTCCTGGCCTGTGTTCCCAGCCTTGGGAAGTCGCTCCCATGACCTG
GCCTTGCTCATCTGCTTCCCTTGTGCAAACAGGTGTTCGACGCCATCATGAACTTCAGGAAGGAGGAGACAGCCAAGCTGATCGAGA
AGCTGGACATCAAGCTGGACAGCGAGGACAAAGACAAGGAGGGCAAGCCATTGCTCAAGGCTGTGATGCGCCGCTGGCTGCCTGCA
GGGGACGCCCTCCTGCAGATGATCACCATCCACTTACCATCCCCCGTCACTGCACAGAAGTACCGTTGTGAGCTGCTATACGAGGGG
CCTCCCGACGATGAGGCCGCCATGGGTATGTACTGTAGGGGCTCCTGGGCTTAGCTCTTGGGTCCTCCAGCCTTGGCAACCAGGTGA
CTCGATTTCCCAACTTTCCATAGGTATTAAGAGCTGCGACCCCAAAGGCCCACTTATGATGTACATTTCCAAGATGGTGCCAACCTCT
GACAAAGGCCGCTTCTATGCCTTTGGTAGAGTGTTCTCTGGGGTGGTGTCCACGGGCCTGAAGGTCCGGATCATGGGCCCCAACTAC
ACGCCTGGGAAGAAAGAGGACCTATACCTGAAGCCTATCCAGAGGTGGGCACTGGCCTTATTTTCCTGAGTCCTGGCTGCTTCCCAG
GAGCACCCTGTTGACAGTTGCCCTCCCTCTAGAACCATTCTGATGATGGGCCGCTACGTGGAGCCGATTGAGGACGTGCCATGTGGA
AACATTGTGGGGCTGGTCGGAGTGGACCAGTTCCTTGTGAAGACGGGGACCATCACTACCTTTGAGCACGCTCACAACATGCGCGTG
ATGAAGTTCAGCGTCAGCCCTGTCGTCCGTGTGGCAGTGGAGGCCAAGAACCCAGCTGACCTGCCCAAGCTGGTGGAGGGGCTGAA
GCGGCTGGCTAAGTCTGACCCTATGGTGCAGGTGTGCCACTAGGTCTTGGCTTATTGTGGTTGGAGAGGGGGAACACCTGGCAAAAG
CAGGGGAGGGCTGAGGCTAGGCCTGTATTGGCTGTTGTCATAGGGCTGAGCCCTATGCCTGGGGAGGACGGCAGGGTGCTGACCAT
TGTTCCCACAGTGCATCATTGAGGAGTCTGGAGAACATATTATTGCTGGCGCCGGTGAGCTGCACCTGGAGATCTGCCTTAAGGACC
TGGAGGAGGACCATGCCTGCATCCCCATCAAGGTGGGCACCAGAATGGGCGTCTCATCCTTCTGCCTATGTGCCTGAGGTGCTGCAC
CATGTGCTGAGGGTTCTATGCAGGCCCCCTGGAAGCCCTGTAGTGTTGACAGTTGTGGTCTGTTTCCCTTTCACAGAAATCTGACCCT
GTTGTGTCATATCGGGAGACAGTCAGTGAGGAATCCAACGTGCTGTGTCTGTCCAAGTCCCCCAATAAGCACAACCGGCTGTACATG
AAGGCCAGGCCCTTCCCTGATGGCCTGGCAGAGGACATCGATAAGGGTGAGGTGTCTGCCCGCCAGGAGCTCAAGGCACGTGCCCG
CTACCTGGCCGAAAAGTATGAGTGGGACGTTGCTGAAGCCCGCAAGATCTGGTGCTTTGGCCCTGATGGCACTGGCCCCAACATTCT
CACCGACATCACCAAGGGTGTGCAGTACCTGAATGAGATCAAGGACAGTGTGGTGGCTGGCTTCCAGTGGGCTACTAAGGAGGTAA
GTGACTCAGCATTGGTGACCCTGTGTTCTGAGGGGCTGCTGGTGTCTAACTTCCAGCTCTTCACAGGGCGCTCTCTGTGAGGAAAAC
ATGCGTGGTGTGCGGTTTGATGTTCATGATGTGACCCTGCATGCTGATGCCATTCACCGGGGAGGTGGCCAGATCATCCCCACAGCA
CGCCGCTGCCTGTATGCCAGTGTGCTGACCGCACAGCCCCGCCTCATGGAGCCTATCTATCTGGTGGAGATCCAGGTAACTTGTGTA
TCTTAGGTGGTGTCTGTCTATCCTCCTGCCTCTGCCCACTGCTCACCATGTTCTCTCTTTTTCCTCGAAGTGTCCTGAGCAAGTGGTGG
GTGGCATCTACGGTGTCCTGAACAGGAAGCGTGGCCATGTGTTTGAGGAGTCCCAGGTGGCTGGTACCCCCATGTTTGTGGTCAAGG
CATACCTGCCTGTCAATGAGTCCTTTGGTGAGTGTCTGGTCCCTGGGGTTTGCAGATAACCTGCAAGGCCTGTGTAATATAGCTGCGG
GGTGCAGAACAGAAGCAGATTTCTGTCATCACGGCCAGCTTGCTTAAGGGTGCCAGGAAAGACAGAGCTACAAACTACACAGAGAA
ACCAAAACAAAGGACCCAGGAATCCCTCCTGGGAGGGGTGGCAAGACAGCTAAGCATGTCCCCCCCCCACCCCACCCCCCCCAGGGA
AGTCACCTGAGTCTCCTGTCTTCTGTCTAGGCTTCACCGCTGATCTGCGATCCAACACCGGCGGCCAGGCCTTCCCCCAGTGCGTGTT

TGACCACTGGCAGATCCTGCCTGGGGATCCTTTTGACAACAGCAGCCGCCCCAGCCAAGTGGTAGCTGAGACGCGCAAGCGCAAGG
GCCTGAAAGAGGGCATCCCAGCGCTGGACAACTTCCTGGACAAACTGTAGGCAGCCTGATACTGCCACATGCTGCACAGTGCCCAC
CCATCAGAAGACACCTTGAGACTGTCCCACAGTGCTCCTCTAGAGGCTGCTGGGGCCACCCTGACATCACTCAGCACTCACTTGCTA
CCAATTCTATTTATTTCGGAATTACAAGATAGCGGGAATCTCTCTGCAGGCTGGACTGGCAGGCTGTGGGGTGGGCGGGACACGGCT
CTTAACATTTTCAGAGGGAAACGCGCAGATGTCCAAAAGTCTAAATAAATGCATTCAGAGGTTTTTGGGGTCCATGGCCAAGTGGAG
TTCCCCCAGAGGGGGAGGTGGGGTAAGTGCCTCCAGGAAGGCAGGCAGCCTGCCTTAGACTTGCAGCCCGGCTGTGGGAATGAATC
ATTGGAGTAATAAACTACAGTGGTTGATCCACATGTGCTCGCAACTCTGTTTTGTACAGCGCACGCTTTCCTGGTTTTCAGGGTATCT
GGCTACCCTTAGGTGGCCTGCGGTGATTCACACATAACACACACACTGCACCCCGTGCGACAGAATGGGTTTTATTGTGAGTTCACA
GTACTGCCTCAGTCCATTCCACAAACTTGAATCACTCCATAGACCTGCACATCAGGGGTTTGGTCTGCGCATACCCGGCTCCAGGTTA
TTGGCTTTCAGCCTCTCGCGTTAGCCAATCATCAGCCAGGAGCCCAATAATGATGTGCTCCACAAGGGCGGTGTCGAGCCTTCCTGT
GAATCTGTCAGGCTGGCCAATGAGCACTGGACTCTCGCCTAGCCTTGGGTGATGACGTCACTAGTCAACGTCCGCTTCCGCTGAGAG
GTCAGATGCTAACCAATGAGCGTGTGCATTCAATTTCCGGTTAAAGTCGTAGTCCAATGAGCGCTGGTTTTCGGGTTTTGCGGAGGT
GTGCCCCTGCGCTGTCAGCGCATGCGTAGATGCCGTCTCAGGATTTGTACCGGAGGTTCTCGCGGGGGCCTCGCTGGCACCGGGCGG
GGACTCGATGGCTGAGGACGGTACGAATGGG

Gn.Ex	Type	S	.Begin	...End	.Len	Fr	Ph	I/Ac	Do/T	CodRg	P....	Tscr..
1.00	Prom	+	580	619	40							1.19
1.01	Init	+	697	699	3	0	0	117	101	0	0.419	4.13
1.02	Intr	+	1635	1849	215	2	2	104	94	461	0.684	46.34
1.03	Intr	+	2023	2204	182	1	2	104	107	360	0.996	39.53
1.04	Intr	+	2651	2862	212	0	2	92	54	573	0.994	53.46
1.05	Intr	+	3051	3229	179	2	2	100	117	244	0.996	27.73
1.06	Intr	+	3316	3421	106	1	1	92	98	143	0.999	16.42
1.07	Intr	+	3514	3766	253	0	1	105	71	482	0.988	45.74
1.08	Intr	+	3852	4047	196	1	1	89	64	248	0.998	21.49
1.09	Intr	+	4123	4381	259	1	1	75	69	433	0.977	38.10
1.10	Intr	+	4535	4642	108	1	0	70	86	165	0.999	15.48
1.11	Intr	+	4774	5127	354	0	0	97	107	632	0.998	62.04
1.12	Intr	+	5198	5380	183	1	0	56	93	218	0.999	19.60
1.13	Intr	+	5463	5595	133	2	1	82	97	136	0.999	14.62
1.14	Term	+	5859	6052	194	1	2	96	48	341	0.739	28.81
1.15	PlyA	+	6306	6311	6							1.05

>miR-eGFP-6th_int_eEF2 (used in Exp. No.2)
GACAAAAAAAGTTTCATGAGAGCGTGGTGTGTTGGCACAGTCTTTTAGTCCGAGCACTGACGCAGTAGATTAAGGCAGATCTTCATG
ATTTGGTGCGGTCCTGGGCTGCATCCAAAGACCTTGTTTCCAAAACAAACAAACGAAAAAGTGTAATAAAAATACAAGCAAGGTTT
TGCAGAAATAAATCTGTGGCCTCAAGGGTCCCTTTCTCCAGCTTTTCCCAGAGGTGTCAAATTACATAACAGTCTCAAACCCAGGAA
ATTGAACTTTGGCACAACCCACTGACCGGTCCGAATTTCAAAGTCTTTTTCCTATTGACCTACAAGGTTTTCAAGAATCATGTTGTAA
GCAACTGTGTTCTGAGGAATCTATGTTTAAAAACCCATCCGTGGATCTTGGCCCAGGGTCCAGAGACTGAGCTAGCCACGCCCCGGC
CGCGCCGCAGCCACTCCCACGGCAGTTCAAGTGTTAAGTCCCAAAGACCGCGCTCTGTGCATGCGCAGACCCGTCCACAGCTGGCTC
CTAGCCAACCCGGCCGGACGAGCACCCGGCGCCGTCACGTGACGCACCCAACCGGCGTCGACCTATAAAAGGCCGGGCGTTGACGT
CAGCGGTCTCTTCCGCCGCAGCCGCCGCCATCGTCGGCGCGCTTCCCTGTTCACCTCTGACTCTGAGAATCCGTCGCCATCCGCCACC
ATGGTGAGTCTCCTCGGCTCCGCTAGACTCGGGGACCGAGACGAATCTCTGGGGCAGCGGGACGTGGCTGTAGCGGGACGCTGAGA
GGGACGGGAGGAAGAGACATGGCTGCCCTGGCCCGGGCGGCAGGACGTGGTCGGGCCGCGGCGCCATATCTGCGCGTCCCTGAGG
GCCTTGGGAGTGTCAACTGCCGAGGTCGGGGTGTTTTCTTGAAGTCCTTCAACTCCCCGCGGCCGCCGGGGTGACTGCGGGAGGGGT
TGTGCTTGGTGATGTGGCAGCGGGCAAAGCGCCGTCCCCGCGCCCCTGGTGACGGGCGGAGGGTGTCCTCGGGAGGTGACAGCCTG
TAGGGCTGGCTTCCTTGGACACCTCCAGTGGGCTGAACGCCTTCCGGGCCCTTTCCGGTAGCCCCCGTCTCTGTTTTCTATCTGAGTT
CACACGTGAGCACCGGTCCCCATAATCTAAGAAAGTGGCTCACTGGGCCTAGTGGCGCATTGTGGCCTTTGATCCGGGCTTTGACCT
TGGCGCACAGCACCCAGTGGTTTGGGGAAGAGGTGTGTGTAGCAGAGGAGGTTTTTTCGTGCTTTGGTCCCAATCAATCCGGCATCT
TTGCAGTGCCGAGGTGGCCGTGCACCTTGGCTTTGAATTCTTGTGCTGAGGTTATGTGACTTGAGCCTCAAGATAGGGTCTTCTAGCA
CAGGCTTGCTCTTAAGTGTCGCAGTTGTCGGTTTCGGCGTTTGTTTAGAGCTGTGGACACATCTGTGAACTTTTGATGCTTATTTCAG
AGGTCCTGGGTTGTACGTTTGAGTCACACTGTGAGCTCAGCTCCAATCTTGGGCCGACATCTGGTTCCTGCCCCTGCTGTGGGGTGCT
ATTGACCCACCGATGCCTGCCAAGTTGGGTTCCCAGAATCAGCCTGGCTGCCCATCCCCCCACCACAGGTGAACTTCACAGTAGATC
AGATCCGTGCCATCATGGACAAGAAAGCCAACATCCGGAACATGTCAGTCATCGCCCATGTGGACCACGGCAAGTCCACGCTGACC
GACTCCCTTGTGTGCAAGGCTGGCATCATTGCCTCTGCCCGAGCTGGGGAGACGCGCTTCACTGACACTCGCAAGGATGAGCAGGAG
CGCTGCATCACAATCAAATCCACGTGAGTGAGGGGACAGCCCCGAGGGGTTGTGCTCTGGGTGTCACTCGGGTGTGTTTGCTGCCAG
TGGACAAATAGTGGCGTGTTTAGGGTCCTCTCTGTGACATGCTTGTGTGTGCTGCCTAGCCTGCCTTGGTCAGATGCTGAATGGACTG
TTGACCTCTTCCTCTCCGTAGCGCCATCTCCCTCTTCTACGAGCTCTCTGAGAACGACCTGAACTTCATTAAGCAGAGCAAGGATGGC
TCGGGCTTCCTCATCAACCTCATCGACTCTCCAGGCCATGTGGACTTCTCTTCAGAGGTGACAGCTGCCTTGCGTGTCACCGATGGAG
CTCTGGTGGTGGTGGACTGTGTGTCTGGTAAGCCTTGGGTGGAGCTGTAACAGACAGTTGCTGGTGTTTTCATGGCCCAGTACCAC
ATTTTTGGGGGTGTAAATTGAAATCATATTCATTTTAAGGCAAGGATGAATTCTGCTCTAATTTGAAATTGTATGTGCCAGTCCCCAT

GTCAGGGCAGGGAGATTGTGAAGTCCACCTAAGTGTGGGGCCAGTTTCCTATAATTTCAGTGCTCTGAGGCAGAGGTGCATGGGTCT
CTGGAGGTGGGAGGGGCTGAGTGGCATAGGGCCTGGTACACTGGACACACTTGGAGATTTAGCGCCTGGGCATCCATACCGTGTAC
CTTGGAGAGTGCTTGTGTACATTTCACAGACAAAACATACGTGGTCTTCAGGCCTTGAACTGTCAGTGGTTTAGAGTACTTGAGAGT
TAAGGGTAAACACTTAGTCCTTTTCACCCTGTCCTCAGGCGTGTGTGTGCAGACAGAAACTGTGCTGCGCCAGGCCATCGCTGAACG
CATCAAGCCCGTCCTGATGATGAACAAGATGGACCGGGCCCTGCTGGAGCTGCAGCTGGAGCCCGAGGAGCTCTACCAGACCTTCC
AGCGCATCGTGGAGAACGTCAACGTCATCATCTCTACCTACGGCGAGGGCGAGAGTGGGCCCATGGGCAATATCATGGTGCGAAGC
TCTTCTATGGGGGGCGGTCAGGGCCACGCTCCCTGGGAATCTCTGACCCACCATGATGACGGAAATTCTTCACTTTGACCTGATGTCT
GTTGAAGAAACTCAGTGTCTGAGACTGTGGGCTTGTGGCTGGGCTGGCTGCTGACCATCTTGCCGGGCTAACTGCTGCTCTCCTCCCT
CAGATTGACCCCGTCCTGGGCACCGTAGGCTTTGGTTCTGGCCTGCATGGCTGGGCCTTCACCCTGAAGCAGTTTGCGGAGATGTAT
GTGGCCAAGTTTGCAGCCAAGGGTGAGGGCCAGCTGAGCGCAGCCGAGCGTGCCAAGAAAGTAGAGGACATGATGAAGAAGCTGT
GGGGAGACCGGTGAGTACATGGCTGTGTGACTGTCTCTACCCCTGCTACTTGTGGACAATGCCTGCCTGCCGATACTATGTGACTGC
TCTCTACAGGTACTTTGATCCGGCCAATGGCAAGTTCAGTAAGTCAGCCAATAGCCCAGATGGGAAAAAACTTCCCCGCACCTTCTG
CCAGCTCATCCTGGACCCCATCTTCAAGGTGGGTAAGCTGTGGTCCTGGCCTGTGTTCCCAGCTAGTGAGTCGACCAGTGGATCCTG
GAGGCTTGCTGAAGGCTGTATGCTGATGAACTTCAGGGTCAGCTTGGTTTTGGCCACTGACTGACCAAGCTGACTGAAGTTCATCAG
GACACAAGGCCTGTTACTAGCACTCACATGGAACAAATGGCCCAGATCCTGGAGGCTTGCTGAAGGCTGTATGCTGTGTAGTTGTAC
TCCAGCTTGTGTTTTGGCCACTGACTGACACAAGCTGGTACAACTACACAGGACACAAGGCCTGTTACTAGCACTCACATGGAACAA
ATGGCCCAGATCTGGCCGCACTCGAGATATCTAGACCCACTTGGGAAGTCGCTCCCATGACCTGGCCTTGCTCATCTGCTTCCCTTGT
GCAAACAGGTGTTCGACGCCATCATGAACTTCAGGAAGGAGGAGACAGCCAAGCTGATCGAGAAGCTGGACATCAAGCTGGACAG
CGAGGACAAAGACAAGGAGGGCAAGCCATTGCTCAAGGCTGTGATGCGCCGCTGGCTGCCTGCAGGGGACGCCCTCCTGCAGATGA
TCACCATCCACTTACCATCCCCCGTCACTGCACAGAAGTACCGTTGTGAGCTGCTATACGAGGGGCCTCCCGACGATGAGGCCGCCA
TGGGTATGTACTGTAGGGGCTCCTGGGCTTAGCTCTTGGGTCCTCCAGCCTTGGCAACCAGGTGACTCGATTTCCCAACTTTCCATAG
GTATTAAGAGCTGCGACCCCAAAGGCCCACTTATGATGTACATTTCCAAGATGGTGCCAACCTCTGACAAAGGCCGCTTCTATGCCT
TTGGTAGAGTGTTCTCTGGGGTGGTGTCCACGGGCCTGAAGGTCCGGATCATGGGCCCCAACTACACGCCTGGGAAGAAAGAGGAC
CTATACCTGAAGCCTATCCAGAGGTGGGCACTGGCCTTATTTTCCTGAGTCCTGGCTGCTTCCCAGGAGCACCCTGTTGACAGTTGCC
CTCCCTCTAGAACCATTCTGATGATGGGCCGCTACGTGGAGCCGATTGAGGACGTGCCATGTGGAAACATTGTGGGGCTGGTCGGAG
TGGACCAGTTCCTTGTGAAGACGGGGACCATCACTACCTTTGAGCACGCTCACAACATGCGCGTGATGAAGTTCAGCGTCAGCCCTG
TCGTCCGTGTGGCAGTGGAGGCCAAGAACCCAGCTGACCTGCCCAAGCTGGTGGAGGGGCTGAAGCGGCTGGCTAAGTCTGACCCT
ATGGTGCAGGTGTGCCACTAGGTCTTGGCTTATTGTGGTTGGAGAGGGGGAACACCTGGCAAAAGCAGGGGAGGGCTGAGGCTAGG
CCTGTATTGGCTGTTGTCATAGGGCTGAGCCCTATGCCTGGGGAGGACGGCAGGGTGCTGACCATTGTTCCCACAGTGCATCATTGA
GGAGTCTGGAGAACATATTATTGCTGGCGCCGGTGAGCTGCACCTGGAGATCTGCCTTAAGGACCTGGAGGAGGACCATGCCTGCA
TCCCCATCAAGGTGGGCACCAGAATGGGCGTCTCATCCTTCTGCCTATGTGCCTGAGGTGCTGCACCATGTGCTGAGGGTTCTATGC
AGGCCCCCTGGAAGCCCTGTAGTGTTGACAGTTGTGGTCTGTTTCCCTTTCACAGAAATCTGACCCTGTTGTGTCATATCGGGAGACA
GTCAGTGAGGAATCCAACGTGCTGTGTCTGTCCAAGTCCCCCAATAAGCACAACCGGCTGTACATGAAGGCCAGGCCCTTCCCTGAT
GGCCTGGCAGAGGACATCGATAAGGGTGAGGTGTCTGCCCGCCAGGAGCTCAAGGCACGTGCCCGCTACCTGGCCGAAAAGTATGA
GTGGGACGTTGCTGAAGCCCGCAAGATCTGGTGCTTTGGCCCTGATGGCACTGGCCCCAACATTCTCACCGACATCACCAAGGGTGT
GCAGTACCTGAATGAGATCAAGGACAGTGTGGTGGCTGGCTTCCAGTGGGCTACTAAGGAGGTAAGTGACTCAGCATTGGTGACCC
TGTGTTCTGAGGGGCTGCTGGTGTCTAACTTCCAGCTCTTCACAGGGCGCTCTCTGTGAGGAAAACATGCGTGGTGTGCGGTTTGATG
TTCATGATGTGACCCTGCATGCTGATGCCATTCACCGGGGAGGTGGCCAGATCATCCCCACAGCACGCCGCTGCCTGTATGCCAGTG
TGCTGACCGCACAGCCCCGCCTCATGGAGCCTATCTATCTGGTGGAGATCCAGGTAACTTGTGTATCTTAGGTGGTGTCTGTCTATCC
TCCTGCCTCTGCCCACTGCTCACCATGTTCTCTCTTTTTCCTCGAAGTGTCCTGAGCAAGTGGTGGGTGGCATCTACGGTGTCCTGAA
CAGGAAGCGTGGCCATGTGTTTGAGGAGTCCCAGGTGGCTGGTACCCCCATGTTTGTGGTCAAGGCATACCTGCCTGTCAATGAGTC
CTTTGGTGAGTGTCTGGTCCCTGGGGTTTGCAGATAACCTGCAAGGCCTGTGTAATATAGCTGCGGGGTGCAGAACAGAAGCAGATT
TCTGTCATCACGGCCAGCTTGCTTAAGGGTGCCAGGAAAGACAGAGCTACAAACTACACAGAGAAACCAAAACAAAGGACCCAGG
AATCCCTCCTGGGAGGGGTGGCAAGACAGCTAAGCATGTCCCCCCCCCACCCCACCCCCCCAGGGAAGTCACCTGAGTCTCCTGTCT
TCTGTCTAGGCTTCACCGCTGATCTGCGATCCAACACCGGCGGCCAGGCCTTCCCCCAGTGCGTGTTTGACCACTGGCAGATCCTGCC
TGGGGATCCTTTTGACAACAGCAGCCGCCCCAGCCAAGTGGTAGCTGAGACGCGCAAGCGCAAGGGCCTGAAAGAGGGCATCCCAG
CGCTGGACAACTTCCTGGACAAACTGTAGGCAGCCTGATACTGCCACATGCTGCACAGTGCCCACCCATCAGAAGACACCTTGAGAC
TGTCCCACAGTGCTCCTCTAGAGGCTGCTGGGGCCACCCTGACATCACTCAGCACTCACTTGCTACCAATTCTATTTATTTCGGAATT
ACAAGATAGCGGGAATCTCTCTGCAGGCTGGACTGGCAGGCTGTGGGGTGGGCGGGACACGGCTCTTAACATTTTCAGAGGGAAAC
GCGCAGATGTCCAAAAGTCTAAATAAATGCATTCAGAGGTTTTTGGGGTCCATGGCCAAGTGGAGTTCCCCCAGAGGGGGAGGTGG
GGTAAGTGCCTCCAGGAAGGCAGGCAGCCTGCCTTAGACTTGCAGCCCGGCTGTGGGAATGAATCATTGGAGTAATAAACTACAGT
GGTTGATCCACATGTGCTCGCAACTCTGTTTTGTACAGCGCACGCTTTCCTGGTTTTCAGGGTATCTGGCTACCCTTAGGTGGCCTGC
GGTGATTCACACATAACACACACACTGCACCCCGTGCGACAGAATGGGTTTTATTGTGAGTTCACAGTACTGCCTCAGTCCATTCCA
CAAACTTGAATCACTCCATAGACCTGCACATCAGGGGTTTGGTCTGCGCATACCCGGCTCCAGGTTATTGGCTTTCAGCCTCTCGCGT
TAGCCAATCATCAGCCAGGAGCCCAATAATGATGTGCTCCACAAGGGCGGTGTCGAGCCTTCCTGTGAATCTGTCAGGCTGGCCAAT
GAGCACTGGACTCTCGCCTAGCCTTGGGTGATGACGTCACTAGTCAACGTCCGCTTCCGCTGAGAGGTCAGATGCTAACCAATGAGC
GTGTGCATTCAATTTCCGGTTAAAGTCGTAGTCCAATGAGCGCTGGTTTTCGGGTTTTGCGGAGGTGTGCCCCTGCGCTGTCAGCGCA
TGCGTAGATGCCGTCTCAGGATTTGTACCGGAGGTTCTCGCGGGGGCCTCGCTGGCACCGGGCGGGGACTCGATGGCTGAGGACGG
TACGAATGGG

Gn.Ex	Type	S	.Begin	...End	.Len	Fr	Ph	I/Ac	Do/T	CodRg	P....	Tscr..
1.00	Prom	+	580	619	40							1.19
1.01	Init	+	697	699	3	0	0	117	101	0	0.419	4.13
1.02	Intr	+	1635	1849	215	2	2	104	94	461	0.684	46.34
1.03	Intr	+	2023	2204	182	1	2	104	107	360	0.996	39.53
1.04	Intr	+	2651	2862	212	0	2	92	54	573	0.994	53.46
1.05	Intr	+	3051	3229	179	2	2	100	117	244	0.996	27.73
1.06	Intr	+	3316	3421	106	1	1	92	98	143	0.965	16.42
1.07	Intr	+	3838	4090	253	0	1	105	71	482	0.988	45.74
1.08	Intr	+	4176	4371	196	1	1	89	64	248	0.998	21.49
1.09	Intr	+	4447	4705	259	1	1	75	69	433	0.977	38.10
1.10	Intr	+	4859	4966	108	1	0	70	86	165	0.999	15.48
1.11	Intr	+	5098	5451	354	0	0	97	107	632	0.998	62.04
1.12	Intr	+	5522	5704	183	1	0	56	93	218	0.999	19.60
1.13	Intr	+	5787	5919	133	2	1	82	97	136	0.999	14.62
1.14	Term	+	6183	6376	194	1	2	96	48	341	0.739	28.81
1.15	PlyA	+	6630	6635	6							1.05

>miR-Otx2_546-6th_int_eEF2 (used in Exp. No.4)
GACAAAAAAAGTTTCATGAGAGCGTGGTGTGTTGGCACAGTCTTTTAGTCCGAGCACTGACGCAGTAGATTAAGGCAGATCTTCATG
ATTTGGTGCGGTCCTGGGCTGCATCCAAAGACCTTGTTTCCAAAACAAACAAACGAAAAAGTGTAATAAAAATACAAGCAAGGTTT
TGCAGAAATAAATCTGTGGCCTCAAGGGTCCCTTTCTCCAGCTTTTCCCAGAGGTGTCAAATTACATAACAGTCTCAAACCCAGGAA
ATTGAACTTTGGCACAACCCACTGACCGGTCCGAATTTCAAAGTCTTTTTCCTATTGACCTACAAGGTTTTCAAGAATCATGTTGTAA
GCAACTGTGTTCTGAGGAATCTATGTTTAAAAACCCATCCGTGGATCTTGGCCCAGGGTCCAGAGACTGAGCTAGCCACGCCCCGGC
CGCGCCGCAGCCACTCCCACGGCAGTTCAAGTGTTAAGTCCCAAAGACCGCGCTCTGTGCATGCGCAGACCCGTCCACAGCTGGCTC
CTAGCCAACCCGGCCGGACGAGCACCCGGCGCCGTCACGTGACGCACCCAACCGGCGTCGACCTATAAAAGGCCGGGCGTTGACGT
CAGCGGTCTCTTCCGCCGCAGCCGCCGCCATCGTCGGCGCGCTTCCCTGTTCACCTCTGACTCTGAGAATCCGTCGCCATCCGCCACC
ATGGTGAGTCTCCTCGGCTCCGCTAGACTCGGGGACCGAGACGAATCTCTGGGGCAGCGGGACGTGGCTGTAGCGGGACGCTGAGA
GGGACGGGAGGAAGAGACATGGCTGCCCTGGCCCGGGCGGCAGGACGTGGTCGGGCCGCGGCGCCATATCTGCGCGTCCCTGAGG
GCCTTGGGAGTGTCAACTGCCGAGGTCGGGGTGTTTTCTTGAAGTCCTTCAACTCCCCGCGGCCGCCGGGGTGACTGCGGGAGGGGT
TGTGCTTGGTGATGTGGCAGCGGGCAAAGCGCCGTCCCCGCGCCCCTGGTGACGGGCGGAGGGTGTCCTCGGGAGGTGACAGCCTG
TAGGGCTGGCTTCCTTGGACACCTCCAGTGGGCTGAACGCCTTCCGGGCCCTTTCCGGTAGCCCCCGTCTCTGTTTTCTATCTGAGTT
CACACGTGAGCACCGGTCCCCATAATCTAAGAAAGTGGCTCACTGGGCCTAGTGGCGCATTGTGGCCTTTGATCCGGGCTTTGACCT
TGGCGCACAGCACCCAGTGGTTTGGGGAAGAGGTGTGTGTAGCAGAGGAGGTTTTTTCGTGCTTTGGTCCCAATCAATCCGGCATCT
TTGCAGTGCCGAGGTGGCCGTGCACCTTGGCTTTGAATTCTTGTGCTGAGGTTATGTGACTTGAGCCTCAAGATAGGGTCTTCTAGCA
CAGGCTTGCTCTTAAGTGTCGCAGTTGTCGGTTTCGGCGTTTGTTTAGAGCTGTGGACACATCTGTGAACTTTTGATGCTTATTTCAG
AGGTCCTGGGTTGTACGTTTGAGTCACACTGTGAGCTCAGCTCCAATCTTGGGCCGACATCTGGTTCCTGCCCCTGCTGTGGGGTGCT
ATTGACCCACCGATGCCTGCCAAGTTGGGTTCCCAGAATCAGCCTGGCTGCCCATCCCCCCACCACAGGTGAACTTCACAGTAGATC
AGATCCGTGCCATCATGGACAAGAAAGCCAACATCCGGAACATGTCAGTCATCGCCCATGTGGACCACGGCAAGTCCACGCTGACC
GACTCCCTTGTGTGCAAGGCTGGCATCATTGCCTCTGCCCGAGCTGGGGAGACGCGCTTCACTGACACTCGCAAGGATGAGCAGGAG
CGCTGCATCACAATCAAATCCACGTGAGTGAGGGGACAGCCCCGAGGGGTTGTGCTCTGGGTGTCACTCGGGTGTGTTTGCTGCCAG
TGGACAAATAGTGGCGTGTTTAGGGTCCTCTCTGTGACATGCTTGTGTGTGCTGCCTAGCCTGCCTTGGTCAGATGCTGAATGGACTG
TTGACCTCTTCCTCTCCGTAGCGCCATCTCCCTCTTCTACGAGCTCTCTGAGAACGACCTGAACTTCATTAAGCAGAGCAAGGATGGC
TCGGGCTTCCTCATCAACCTCATCGACTCTCCAGGCCATGTGGACTTCTCTTCAGAGGTGACAGCTGCCTTGCGTGTCACCGATGGAG
CTCTGGTGGTGGTGGACTGTGTGTCTGGTAAGCCTTGGGTGGGAGCTGTAACAGACAGTTGCTGGTGTTTTCATGGCCCAGTACCAC
ATTTTTGGGGGTGTAAATTGAAATCATATTCATTTTAAGGCAAGGATGAATTCTGCTCTAATTTGAAATTGTATGTGCCAGTCCCCAT
GTCAGGGCAGGGAGATTGTGAAGTCCACCTAAGTGTGGGGCCAGTTTCCTATAATTTCAGTGCTCTGAGGCAGAGGTGCATGGGTCT
CTGGAGGTGGGAGGGGCTGAGTGGCATAGGGCCTGGTACACTGGACACACTTGGAGATTTAGCGCCTGGGCATCCATACCGTGTAC
CTTGGAGAGTGCTTGTGTACATTTCACAGACAAAACATACGTGGTCTTCAGGCCTTGAACTGTCAGTGGTTTAGAGTACTTGAGAGT
TAAGGGTAAACACTTAGTCCTTTTCACCCTGTCCTCAGGCGTGTGTGTGCAGACAGAAACTGTGCTGCGCCAGGCCATCGCTGAACG
CATCAAGCCCGTCCTGATGATGAACAAGATGGACCGGGCCCTGCTGGAGCTGCAGCTGGAGCCCGAGGAGCTCTACCAGACCTTCC
AGCGCATCGTGGAGAACGTCAACGTCATCATCTCTACCTACGGCGAGGGCGAGAGTGGGCCCATGGGCAATATCATGGTGCGAAGC
TCTTCTATGGGGGGCGGTCAGGGCCACGCTCCCTGGGAATCTCTGACCCACCATGATGACGGAAATTCTTCACTTTGACCTGATGTCT
GTTGAAGAAACTCAGTGTCTGAGACTGTGGGCTTGTGGCTGGGCTGGCTGCTGACCATCTTGCCGGGCTAACTGCTGCTCTCCTCCCT
CAGATTGACCCCGTCCTGGGCACCGTAGGCTTTGGTTCTGGCCTGCATGGCTGGGCCTTCACCCTGAAGCAGTTTGCGGAGATGTAT
GTGGCCAAGTTTGCAGCCAAGGGTGAGGGCCAGCTGAGCGCAGCCGAGCGTGCCAAGAAAGTAGAGGACATGATGAAGAAGCTGT
GGGGAGACCGGTGAGTACATGGCTGTGTGACTGTCTCTACCCCTGCTACTTGTGGACAATGCCTGCCTGCCGATACTATGTGACTGC
TCTCTACAGGTACTTTGATCCGGCCAATGGCAAGTTCAGTAAGTCAGCCAATAGCCCAGATGGGAAAAAACTTCCCCGCACCTTCTG
CCAGCTCATCCTGGACCCCATCTTCAAGGTGGGTAAGCTGTGGTCCTGGCCTGTGTTCCCAGCTAGTGAGTCGACCAGTGGATCCTG
GAGGCTTGCTGAAGGCTGTATGCTGTAGCCTTGACTATAACCTGAAGTTTGGCCACTGACTGACTTCAGGTTAGTCAAGGCTACAG

GACACAAGGCCTGTTACTAGCACTCACATGGAACAAATGGCCCAGATCTGGCCGCACTCGAGATATCTAGACCCACTTGGGAAGTC
GCTCCCATGACCTGGCCTTGCTCATCTGCTTCCCTTGTGCAAACAGGTGTTCGACGCCATCATGAACTTCAGGAAGGAGGAGACAGC
CAAGCTGATCGAGAAGCTGGACATCAAGCTGGACAGCGAGGACAAAGACAAGGAGGGCAAGCCATTGCTCAAGGCTGTGATGCGC
CGCTGGCTGCCTGCAGGGGACGCCCTCCTGCAGATGATCACCATCCACTTACCATCCCCCGTCACTGCACAGAAGTACCGTTGTGAG
CTGCTATACGAGGGGCCTCCCGACGATGAGGCCGCCATGGGTATGTACTGTAGGGGCTCCTGGGCTTAGCTCTTGGGTCCTCCAGCC
TTGGCAACCAGGTGACTCGATTTCCCAACTTTCCATAGGTATTAAGAGCTGCGACCCCAAAGGCCCACTTATGATGTACATTTCCAA
GATGGTGCCAACCTCTGACAAAGGCCGCTTCTATGCCTTTGGTAGAGTGTTCTCTGGGGTGGTGTCCACGGGCCTGAAGGTCCGGAT
CATGGGCCCCAACTACACGCCTGGGAAGAAAGAGGACCTATACCTGAAGCCTATCCAGAGGTGGGCACTGGCCTTATTTCCTGAGT
CCTGGCTGCTTCCCAGGAGCACCCTGTTGACAGTTGCCCTCCCTCTAGAACCATTCTGATGATGGGCCGCTACGTGGAGCCGATTGA
GGACGTGCCATGTGGAAACATTGTGGGGCTGGTCGGAGTGGACCAGTTCCTTGTGAAGACGGGGACCATCACTACCTTTGAGCACG
CTCACAACATGCGCGTGATGAAGTTCAGCGTCAGCCCTGTCGTCCGTGTGGCAGTGGAGGCCAAGAACCCAGCTGACCTGCCCAAG
CTGGTGGAGGGGCTGAAGCGGCTGGCTAAGTCTGACCCTATGGTGCAGGTGTGCCACTAGGTCTTGGCTTATTGTGGTTGGAGAGGG
GGAACACCTGGCAAAAGCAGGGGAGGGCTGAGGCTAGGCCTGTATTGGCTGTTGTCATAGGGCTGAGCCCTATGCCTGGGGAGGAC
GGCAGGGTGCTGACCATTGTTCCCACAGTGCATCATTGAGGAGTCTGGAGAACATATTATTGCTGGCGCCGGTGAGCTGCACCTGGA
GATCTGCCTTAAGGACCTGGAGGAGGACCATGCCTGCATCCCCATCAAGGTGGGCACCAGAATGGGCGTCTCATCCTTCTGCCTATG
TGCCTGAGGTGCTGCACCATGTGCTGAGGGTTCTATGCAGGCCCCCTGGAAGCCCTGTAGTGTTGACAGTTGTGGTCTGTTTCCCTTT
CACAGAAATCTGACCCTGTTGTGTCATATCGGGAGACAGTCAGTGAGGAATCCAACGTGCTGTGTCTGTCCAAGTCCCCCAATAAGC
ACAACCGGCTGTACATGAAGGCCAGGCCCTTCCCTGATGGCCTGGCAGAGGACATCGATAAGGGTGAGGTGTCTGCCCGCCAGGAG
CTCAAGGCACGTGCCCGCTACCTGGCCGAAAAGTATGAGTGGGACGTTGCTGAAGCCCGCAAGATCTGGTGCTTTGGCCCTGATGGC
ACTGGCCCCAACATTCTCACCGACATCACCAAGGGTGTGCAGTACCTGAATGAGATCAAGGACAGTGTGGTGGCTGGCTTCCAGTGG
GCTACTAAGGAGGTAAGTGACTCAGCATTGGTGACCCTGTGTTCTGAGGGGCTGCTGGTGTCTAACTTCCAGCTCTTCACAGGGCGC
TCTCTGTGAGGAAAACATGCGTGGTGTGCGGTTTGATGTTCATGATGTGACCCTGCATGCTGATGCCATTCACCGGGGAGGTGGCCA
GATCATCCCCACAGCACGCCGCTGCCTGTATGCCAGTGTGCTGACCGCACAGCCCCGCCTCATGGAGCCTATCTATCTGGTGGAGAT
CCAGGTAACTTGTGTATCTTAGGTGGTGTCTGTCTATCCTCCTGCCTCTGCCCACTGCTCACCATGTTCTCTCTTTTTCCTCGAAGTGT
CCTGAGCAAGTGGTGGGTGGCATCTACGGTGTCCTGAACAGGAAGCGTGGCCATGTGTTTGAGGAGTCCCAGGTGGCTGGTACCCCC
ATGTTTGTGGTCAAGGCATACCTGCCTGTCAATGAGTCCTTTGGTGAGTGTCTGGTCCCTGGGGTTTGCAGATAACCTGCAAGGCCTG
TGTAATATAGCTGCGGGGTGCAGAACAGAAGCAGATTTCTGTCATCACGGCCAGCTTGCTTAAGGGTGCCAGGAAAGACAGAGCTA
CAAACTACACAGAGAAACCAAAACAAAGGACCCAGGAATCCCTCCTGGGAGGGGTGGCAAGACAGCTAAGCATGTCCCCCCCCCA
CCCCACCCCCCCAGGGAAGTCACCTGAGTCTCCTGTCTTCTGTCTAGGCTTCACCGCTGATCTGCGATCCAACACCGGCGGCCAGGC
CTTCCCCCAGTGCGTGTTTGACCACTGGCAGATCCTGCCTGGGGATCCTTTTGACAACAGCAGCCGCCCCAGCCAAGTGGTAGCTGA
GACGCGCAAGCGCAAGGGCCTGAAAGAGGGCATCCCAGCGCTGGACAACTTCCTGGACAAACTGTAGGCAGCCTGATACTGCCACA
TGCTGCACAGTGCCCACCCATCAGAAGACACCTTGAGACTGTCCCACAGTGCTCCTCTAGAGGCTGCTGGGGCCACCCTGACATCAC
TCAGCACTCACTTGCTACCAATTCTATTTATTTCGGAATTACAAGATAGCGGGAATCTCTCTGCAGGCTGGACTGGCAGGCTGTGGG
GTGGGCGGGACACGGCTCTTAACATTTTCAGAGGGAAACGCGCAGATGTCCAAAAGTCTAAATAAATGCATTCAGAGGTTTTTGGG
GTCCATGGCCAAGTGGAGTTCCCCCAGAGGGGGAGGTGGGGTAAGTGCCTCCAGGAAGGCAGGCAGCCTGCCTTAGACTTGCAGCC
CGGCTGTGGGAATGAATCATTGGAGTAATAAACTACAGTGGTTGATCCACATGTGCTCGCAACTCTGTTTTGTACAGCGCACGCTTT
CCTGGTTTTCAGGGTATCTGGCTACCCTTAGGTGGCCTGCGGTGATTCACACATAACACACACACTGCACCCCGTGCGACAGAATGG
GTTTTATTGTGAGTTCACAGTACTGCCTCAGTCCATTCCACAAACTTGAATCACTCCATAGACCTGCACATCAGGGGTTTGGTCTGCG
CATACCCGGCTCCAGGTTATTGGCTTTCAGCCTCTCGCGTTAGCCAATCATCAGCCAGGAGCCCAATAATGATGTGCTCCACAAGGG
CGGTGTCGAGCCTTCCTGTGAATCTGTCAGGCTGGCCAATGAGCACTGGACTCTCGCCTAGCCTTGGGTGATGACGTCACTAGTCAA
CGTCCGCTTCCGCTGAGAGGTCAGATGCTAACCAATGAGCGTGTGCATTCAATTTCCGGTTAAAGTCGTAGTCCAATGAGCGCTGGT
TTTCGGGTTTTGCGGAGGTGTGCCCCTGCGCTGTCAGCGCATGCGTAGATGCCGTCTCAGGATTTGTACCGGAGGTTCTCGCGGGGG
CCTCGCTGGCACCGGGCGGGGACTCGATGGCTGAGGACGGTACGAATGGG

Gn.Ex	Type	S	.Begin	...End	.Len	Fr	Ph	I/Ac	Do/T	CodRg	P....	Tscr..
-----	----	-	------	------	----	--	--	----	----	-----	-----	------
1.00	Prom	+	580	619	40							1.19
1.01	Init	+	697	699	3	0	0	117	101	0	0.419	4.13
1.02	Intr	+	1635	1849	215	2	2	104	94	461	0.684	46.34
1.03	Intr	+	2023	2204	182	1	2	104	107	360	0.996	39.53
1.04	Intr	+	2651	2862	212	0	2	92	54	573	0.994	53.46
1.05	Intr	+	3051	3229	179	2	2	100	117	244	0.996	27.73
1.06	Intr	+	3316	3421	106	1	1	92	98	143	0.999	16.42
1.07	Intr	+	3700	3952	253	0	1	105	71	482	0.988	45.74
1.08	Intr	+	4038	4233	196	1	1	89	64	248	0.998	21.49
1.09	Intr	+	4309	4567	259	1	1	75	69	433	0.977	38.10
1.10	Intr	+	4721	4828	108	1	0	70	86	165	0.999	15.48
1.11	Intr	+	4960	5313	354	0	0	97	107	632	0.998	62.04
1.12	Intr	+	5384	5566	183	1	0	56	93	218	0.999	19.60
1.13	Intr	+	5649	5781	133	2	1	82	97	136	0.999	14.62
1.14	Term	+	6045	6238	194	1	2	96	48	341	0.739	28.81
1.15	PlyA	+	6492	6497	6							1.05

>cond_miR-eGFP-6th_int_eEF2 (used in Exp. No.5)
GACAAAAAAAGTTTCATGAGAGCGTGGTGTGTTGGCACAGTCTTTTAGTCCGAGCACTGACGCAGTAGATTAAGGCAGATCTTCATG
ATTTGGTGCGGTCCTGGGCTGCATCCAAAGACCTTGTTTCCAAAACAAACAAACGAAAAAGTGTAATAAAAATACAAGCAAGGTTT
TGCAGAAATAAATCTGTGGCCTCAAGGGTCCCTTTCTCCAGCTTTTCCCAGAGGTGTCAAATTACATAACAGTCTCAAACCCAGGAA
ATTGAACTTTGGCACAACCCACTGACCGGTCCGAATTTCAAAGTCTTTTTCCTATTGACCTACAAGGTTTTCAAGAATCATGTTGTAA
GCAACTGTGTTCTGAGGAATCTATGTTTAAAAACCCATCCGTGGATCTTGGCCCAGGGTCCAGAGACTGAGCTAGCCACGCCCCGGC
CGCGCCGCAGCCACTCCCACGGCAGTTCAAGTGTTAAGTCCCAAAGACCGCGCTCTGTGCATGCGCAGACCCGTCCACAGCTGGCTC
CTAGCCAACCCGGCCGGACGAGCACCCGGCGCCGTCACGTGACGCACCCAACCGGCGTCGACCTATAAAAGGCCGGGCGTTGACGT
CAGCGGTCTCTTCCGCCGCAGCCGCCGCCATCGTCGGCGCGCTTCCCTGTTCACCTCTGACTCTGAGAATCCGTCGCCATCCGCCACC
ATGGTGAGTCTCCTCGGCTCCGCTAGACTCGGGGACCGAGACGAATCTCTGGGGCAGCGGGACGTGGCTGTAGCGGGACGCTGAGA
GGGACGGGAGGAAGAGACATGGCTGCCCTGGCCCGGGCGGCAGGACGTGGTCGGGCCGCGGCGCCATATCTGCGCGTCCCTGAGG
GCCTTGGGAGTGTCAACTGCCGAGGTCGGGGTGTTTTCTTGAAGTCCTTCAACTCCCCGCGGCCGCCGGGGTGACTGCGGGAGGGGT
TGTGCTTGGTGATGTGGCAGCGGGCAAAGCGCCGTCCCCGCGCCCCTGGTGACGGGCGGAGGGTGTCCTCGGGAGGTGACAGCCTG
TAGGGCTGGCTTCCTTGGACACCTCCAGTGGGCTGAACGCCTTCCGGGCCCTTTCCGGTAGCCCCCGTCTCTGTTTTCTATCTGAGTT
CACACGTGAGCACCGGTCCCCATAATCTAAGAAAGTGGCTCACTGGGCCTAGTGGCGCATTGTGGCCTTTGATCCGGGCTTTGACCT
TGGCGCACAGCACCCAGTGGTTTGGGGAAGAGGTGTGTGTAGCAGAGGAGGTTTTTTCGTGCTTTGGTCCCAATCAATCCGGCATCT
TTGCAGTGCCGAGGTGGCCGTGCACCTTGGCTTTGAATTCTTGTGCTGAGGTTATGTGACTTGAGCCTCAAGATAGGGTCTTCTAGCA
CAGGCTTGCTCTTAAGTGTCGCAGTTGTCGGTTTCGGCGTTTGTTTAGAGCTGTGGACACATCTGTGAACTTTTGATGCTTATTTCAG
AGGTCCTGGGTTGTACGTTTGAGTCACACTGTGAGCTCAGCTCCAATCTTGGGCCGACATCTGGTTCCTGCCCCTGCTGTGGGGTGCT
ATTGACCCACCGATGCCTGCCAAGTTGGGTTCCCAGAATCAGCCTGGCTGCCCATCCCCCCACCACAGGTGAACTTCACAGTAGATC
AGATCCGTGCCATCATGGACAAGAAAGCCAACATCCGGAACATGTCAGTCATCGCCCATGTGGACCACGGCAAGTCCACGCTGACC
GACTCCCTTGTGTGCAAGGCTGGCATCATTGCCTCTGCCCGAGCTGGGGAGACGCGCTTCACTGACACTCGCAAGGATGAGCAGGAG
CGCTGCATCACAATCAAATCCACGTGAGTGAGGGGACAGCCCCGAGGGGTTGTGCTCTGGGTGTCACTCGGGTGTGTTTGCTGCCAG
TGGACAAATAGTGGCGTGTTTAGGGTCCTCTCTGTGACATGCTTGTGTGTGCTGCCTAGCCTGCCTTGGTCAGATGCTGAATGGACTG
TTGACCTCTTCCTCTCCGTAGCGCCATCTCCCTCTTCTACGAGCTCTCTGAGAACGACCTGAACTTCATTAAGCAGAGCAAGGATGGC
TCGGGCTTCCTCATCAACCTCATCGACTCTCCAGGCCATGTGGACTTCTCTTCAGAGGTGACAGCTGCCTTGCGTGTCACCGATGGAG
CTCTGGTGGTGGTGGACTGTGTGTCTGGTAAGCCTTGGGTGGGAGCTGTAACAGACAGTTGCTGGTGTTTTCATGGCCCAGTACCAC
ATTTTTGGGGGTGTAAATTGAAATCATATTCATTTTAAGGCAAGGATGAATTCTGCTCTAATTTGAAATTGTATGTGCCAGTCCCCAT
GTCAGGGCAGGGAGATTGTGAAGTCCACCTAAGTGTGGGGCCAGTTTCCTATAATTTCAGTGCTCTGAGGCAGAGGTGCATGGGTCT
CTGGAGGTGGGAGGGGCTGAGTGGCATAGGGCCTGGTACACTGGACACACTTGGAGATTTAGCGCCTGGGCATCCATACCGTGTAC
CTTGGAGAGTGCTTGTGTACATTTCACAGACAAAACATACGTGGTCTTCAGGCCTTGAACTGTCAGTGGTTTAGAGTACTTGAGAGT
TAAGGGTAAACACTTAGTCCTTTTCACCCTGTCCTCAGGCGTGTGTGTGCAGACAGAAACTGTGCTGCGCCAGGCCATCGCTGAACG
CATCAAGCCCGTCCTGATGATGAACAAGATGGACCGGGCCCTGCTGGAGCTGCAGCTGGAGCCCGAGGAGCTCTACCAGACCTTCC
AGCGCATCGTGGAGAACGTCAACGTCATCATCTCTACCTACGGCGAGGGCGAGAGTGGGCCCATGGGCAATATCATGGTGCGAAGC
TCTTCTATGGGGGGCGGTCAGGGCCACGCTCCCTGGGAATCTCTGACCCACCATGATGACGGAAATTCTTCACTTTGACCTGATGTCT
GTTGAAGAAACTCAGTGTCTGAGACTGTGGGCTTGTGGCTGGGCTGGCTGCTGACCATCTTGCCGGGCTAACTGCTGCTCTCCTCCCT
CAGATTGACCCCGTCCTGGGCACCGTAGGCTTTGGTTCTGGCCTGCATGGCTGGGCCTTCACCCTGAAGCAGTTTGCGGAGATGTAT
GTGGCCAAGTTTGCAGCCAAGGGTGAGGGCCAGCTGAGCGCAGCCGAGCGTGCCAAGAAAGTAGAGGACATGATGAAGAAGCTGT
GGGGAGACCGGTGAGTACATGGCTGTGTGACTGTCTCTACCCCTGCTACTTGTGGACAATGCCTGCCTGCCGATACTATGTGACTGC
TCTCTACAGGTACTTTGATCCGGCCAATGGCAAGTTCAGTAAGTCAGCCAATAGCCCAGATGGGAAAAAACTTCCCCGCACCTTCTG
CCAGCTCATCCTGGACCCCATCTTCAAGGTGGGTAAGCTGTGGTCCTGGCCTGTGTTCCCAGCaccggtataacttcgtataatgtatgctatacgaataattT
GGGTCTAGATATCTCGAGTGCGGCCAGATCTGGGCCATTTGTTCCATGTGAGTGCTAGTAACAGGCCTTGTGTCCTGTGTAGTTGTAC
CAGCTTGTGTCAGTCAGTGGCCAAAACACAAGCTGGAGTACAACTACACAGCATACAGCCTTCAGCAAGCCTCCAGGATCTGGGCC
ATTTGTTCCATGTGAGTGCTAGTAACAGGCCTTGTGTCCTGATGAACTTCAGTCAGCTTGGTCAGTCAGTGGCCAAAACCAAGCTGA
CCCTGAAGTTCATCAGCATACAGCCTTCAGCAAGCCTCCAGGATCCACTGGTCGACTCACTAataacttcgtatagcatacattatagcaatttatgctagcCT
TGGGAAGTCGCTCCCATGACCTGGCCTTGCTCATCTGCTTCCCTTGTGCAAACAGGTGTTCGACGCCATCATGAACTTCAGGAAGGA
GGAGACAGCCAAGCTGATCGAGAAGCTGGACATCAAGCTGGACAGCGAGGACAAAGACAAGGAGGGCAAGCCATTGCTCAAGGCT
GTGATGCGCCGCTGGCTGCCTGCAGGGGACGCCCTCCTGCAGATGATCACCATCCACTTACCATCCCCCGTCACTGCACAGAAGTAC
CGTTGTGAGCTGCTATACGAGGGGCCTCCCGACGATGAGGCCGCCATGGGTATGTACTGTAGGGGCTCCTGGGCTTAGCTCTTGGGT
CCTCCAGCCTTGGCAACCAGGTGACTCGATTTCCCAACTTTCCATAGGTATTAAGAGCTGCGACCCCAAAGGCCCACTTATGATGTA
CATTTCCAAGATGGTGCCAACCTCTGACAAAGGCCGCTTCTATGCCTTTGGTAGAGTGTTCTCTGGGGTGGTGTCCACGGGCCTGAA
GGTCCGGATCATGGGCCCCAACTACACGCCTGGGAAGAAAGAGGACCTATACCTGAAGCCTATCCAGAGGTGGGCACTGGCCTTAT
TTTCCTGAGTCCTGGCTGCTTCCCAGGAGCACCCTGTTGACAGTTGCCCTCCCTCTAGAACCATTCTGATGATGGGCCGCTACGTGGA
GCCGATTGAGGACGTGCCATGTGGAAACATTGTGGGGCTGGTCGGAGTGGACCAGTTCCTTGTGAAGACGGGGACCATCACTACCTT
TGAGCACGCTCACAACATGCGCGTGATGAAGTTCAGCGTCAGCCCTGTCGTCCGTGTGGCAGTGGAGGCCAAGAACCCAGCTGACC
TGCCCAAGCTGGTGGAGGGGCTGAAGCGGCTGGCTAAGTCTGACCCTATGGTGCAGGTGTGCCACTAGGTCTTGGCTTATTGTGGTT
GGAGAGGGGGAACACCTGGCAAAAGCAGGGGAGGGCTGAGGCTAGGCCTGTATTGGCTGTTGTCATAGGGCTGAGCCCTATGCCTG
GGGAGGACGGCAGGGTGCTGACCATTGTTCCCACAGTGCATCATTGAGGAGTCTGGAGAACATATTATTGCTGGCGCCGGTGAGCT
GCACCTGGAGATCTGCCTTAAGGACCTGGAGGAGGACCATGCCTGCATCCCCATCAAGGTGGGCACCAGAATGGGCGTCTCATCCTT
CTGCCTATGTGCCTGAGGTGCTGCACCATGTGCTGAGGGTTCTATGCAGGCCCCCTGGAAGCCCTGTAGTGTTGACAGTTGTGGTCTG
TTTCCCTTTCACAGAAATCTGACCCTGTTGTGTCATATCGGGAGACAGTCAGTGAGGAATCCAACGTGCTGTGTCTGTCCAAGTCCCC
CAATAAGCACAACCGGCTGTACATGAAGGCCAGGCCCTTCCCTGATGGCCTGGCAGAGGACATCGATAAGGGTGAGGTGTCTGCCC
GCCAGGAGCTCAAGGCACGTGCCCGCTACCTGGCCGAAAAGTATGAGTGGGACGTTGCTGAAGCCCGCAAGATCTGGTGCTTTGGC
CCTGATGGCACTGGCCCCAACATTCTCACCGACATCACCAAGGGTGTGCAGTACCTGAATGAGATCAAGGACAGTGTGGTGGCTGG
CTTCCAGTGGGCTACTAAGGAGGTAAGTGACTCAGCATTGGTGACCCTGTGTTCTGAGGGGCTGCTGGTGTCTAACTTCCAGCTCTTC
ACAGGGCGCTCTCTGTGAGGAAAACATGCGTGGTGTGCGGTTTGATGTTCATGATGTGACCCTGCATGCTGATGCCATTCACCGGGG
AGGTGGCCAGATCATCCCCACAGCACGCCGCTGCCTGTATGCCAGTGTGCTGACCGCACAGCCCCGCCTCATGGAGCCTATCTATCT
GGTGGAGATCCAGGTAACTTGTGTATCTTAGGTGGTGTCTGTCTATCCTCCTGCCTCTGCCCACTGCTCACCATGTTCTCTCTTTTTCC
TCGAAGTGTCCTGAGCAAGTGGTGGGTGGCATCTACGGTGTCCTGAACAGGAAGCGTGGCCATGTGTTTGAGGAGTCCCAGGTGGCT
GGTACCCCCATGTTTGTGGTCAAGGCATACCTGCCTGTCAATGAGTCCTTTGGTGAGTGTCTGGTCCCTGGGGTTTGCAGATAACCTG
CAAGGCCTGTGTAATATAGCTGCGGGGTGCAGAACAGAAGCAGATTTCTGTCATCACGGCCAGCTTGCTTAAGGGTGCCAGGAAAG
ACAGAGCTACAAACTACACAGAGAAACCAAAACAAAGGACCCAGGAATCCCTCCTGGGAGGGGTGGCAAGACAGCTAAGCATGTC
CCCCCCCCACCCCACCCCCCCCAGGGAAGTCACCTGAGTCTCCTGTCTTCTGTCTAGGCTTCACCGCTGATCTGCGATCCAACACCGGC
GGCCAGGCCTTCCCCCAGTGCGTGTTTGACCACTGGCAGATCCTGCCTGGGGATCCTTTTGACAACAGCAGCCGCCCCAGCCAAGTG
GTAGCTGAGACGCGCAAGCGCAAGGGCCTGAAAGAGGGCATCCCAGCGCTGGACAACTTCCTGGACAAACTGTAGGCAGCCTGATA
CTGCCACATGCTGCACAGTGCCCACCCATCAGAAGACACCTTGAGACTGTCCCACAGTGCTCCTCTAGAGGCTGCTGGGGCCACCCT
GACATCACTCAGCACTCACTTGCTACCAATTCTATTTATTTCGGAATTACAAGATAGCGGGAATCTCTCTGCAGGCTGGACTGGCAG
GCTGTGGGGTGGGCGGGACACGGCTCTTAACATTTTCAGAGGGAAACGCGCAGATGTCCAAAAGTCTAAATAAATGCATTCAGAGG

TTTTTGGGGTCCATGGCCAAGTGGAGTTCCCCCAGAGGGGGAGGTGGGGTAAGTGCCTCCAGGAAGGCAGGCAGCCTGCCTTAGAC
TTGCAGCCCGGCTGTGGGAATGAATCATTGGAGTAATAAACTACAGTGGTTGATCCACATGTGCTCGCAACTCTGTTTTGTACAGCG
CACGCTTTCCTGGTTTTCAGGGTATCTGGCTACCCTTAGGTGGCCTGCGGTGATTCACACATAACACACACACTGCACCCCGTGCGAC
AGAATGGGTTTTATTGTGAGTTCACAGTACTGCCTCAGTCCATTCCACAAACTTGAATCACTCCATAGACCTGCACATCAGGGGTTTG
GTCTGCGCATACCCGGCTCCAGGTTATTGGCTTTCAGCCTCTCGCGTTAGCCAATCATCAGCCAGGAGCCCAATAATGATGTGCTCCA
CAAGGGCGGTGTCGAGCCTTCCTGTGAATCTGTCAGGCTGGCCAATGAGCACTGGACTCTCGCCTAGCCTTGGGTGATGACGTCACT
AGTCAACGTCCGCTTCCGCTGAGAGGTCAGATGCTAACCAATGAGCGTGTGCATTCAATTTCCGGTTAAAGTCGTAGTCCAATGAGC
GCTGGTTTTCGGGTTTTGCGGAGGTGTGCCCCTGCGCTGTCAGCGCATGCGTAGATGCCGTCTCAGGATTTGTACCGGAGGTTCTCGC
GGGGGCCTCGCTGGCACCGGGCGGGGACTCGATGGCTGAGGACGGTACGAATGGG

Gn.Ex	Type	S	.Begin	...End	.Len	Fr	Ph	I/Ac	Do/T	CodRg	P....	Tscr..
1.00	Prom	+	580	619	40							1.19
1.01	Init	+	697	699	3	0	0	117	101	0	0.419	4.13
1.02	Intr	+	1635	1849	215	2	2	104	94	461	0.684	46.34
1.03	Intr	+	2023	2204	182	1	2	104	107	360	0.996	39.53
1.04	Intr	+	2651	2862	212	0	2	92	54	573	0.994	53.46
1.05	Intr	+	3051	3229	179	2	2	100	117	244	0.996	27.73
1.06	Intr	+	3316	3421	106	1	1	92	98	143	0.978	16.42
1.07	Intr	+	3918	4170	253	2	1	105	71	482	0.988	45.74
1.08	Intr	+	4256	4451	196	0	1	89	64	248	0.998	21.49
1.09	Intr	+	4527	4785	259	0	1	75	69	433	0.977	38.10
1.10	Intr	+	4939	5046	108	0	0	70	86	165	0.999	15.48
1.11	Intr	+	5178	5531	354	2	0	97	107	632	0.998	62.04
1.12	Intr	+	5602	5784	183	0	0	56	93	218	0.999	19.60
1.13	Intr	+	5867	5999	133	1	1	82	97	136	0.999	14.62
1.14	Term	+	6263	6456	194	0	2	96	48	341	0.739	28.81
1.15	PlyA	+	6710	6715	6							1.05
